# Supplementary material for: Obesity and recovery from acute kidney injury (Ob AKI): a prospective cohort feasibility study
Source: BMJ Open. 2019 Mar 20;9(3):e024033. doi: 10.1136/bmjopen-2018-024033 (PMC6528015; doi:10.1136/bmjopen-2018-024033)
Supplement: Author's manuscript [file bmjopen-2018-024033.draft_revisions.pdf]

BMJ Open is committed to open peer review. As part of this commitment we make the peer review history of every article we publish publicly available.

When an article is published we post the peer reviewers' comments and the authors' responses online. We also post the versions of the paper that were used during peer review. These are the versions that the peer review comments apply to.

The versions of the paper that follow are the versions that were submitted during the peer review process. They are not the versions of record or the final published versions. They should not be cited or distributed as the published version of this manuscript.

BMJ Open is an open access journal and the full, final, typeset and author-corrected version of record of the manuscript is available on our site with no access controls, subscription charges or pay-per-view fees (http://bmjopen.bmj.com).

If you have any questions on BMJ Open's open peer review process please email editorial.bmjopen@bmj.com

BMJ Open

Obesity and recovery from acute kidney injury (Ob AKI): a prospective cohort feasibility study

Journal:	*BMJ Open*
Manuscript ID	bmjopen-2018-024033
Article Type:	Research
Date Submitted by the Author:	20-May-2018
Complete List of Authors:	MacLaughlin, Helen L; King's College Hospital NHS Foundation Trust, Department of Nutrition and Dietetics; King's College London, Department of Nutritional Sciences<br>Blacklock, Rochelle M; King's College Hospital NHS Foundation Trust, Department of Nutrition and Dietetics; King's College London, Department of Nutritional Sciences<br>Wright, Kelly; King's College Hospital NHS Foundation Trust, Department of Nutrition and Dietetics<br>Pot, Gerda ; King's College London, Department of Nutritional Sciences<br>Jayawadene, Satish; King's College Hospital NHS Foundation Trust, Department of Nutrition and Dietetics<br>McIntyre, CW; University of Western Ontario, Division of Nephrology<br>Macdougall, Iain; Kings College Hospital NHS Foundation Trust, Nephrology<br>Selby, Nicholas; University of Nottingham Faculty of Medicine and Health Sciences, Centre for Kidney Research and Innovation; Royal Derby Hospital,
Keywords:	Acute renal failure < NEPHROLOGY, obesity, Chronic renal failure < NEPHROLOGY, NUTRITION & DIETETICS

SCHOLARONE™
Manuscripts

Obesity and recovery from acute kidney injury (Ob AKI): a prospective cohort feasibility study

Helen L MacLaughlin[1,2], Rochelle M Blacklock[1,2], Kelly Wright[3], Gerda Pot[2], Satish Jayawardene[3], Christopher W McIntyre[4], Iain C Macdougall[3], Nicholas M Selby[5]

Institutions

1. Department Nutrition and Dietetics, King's College Hospital, London, United Kingdom
2. Department of Nutritional Sciences, Faculty of Life Sciences and Medicine, King's College London, United Kingdom
3. Department of Renal Medicine, King's College Hospital, London, United Kingdom
4. London Kidney Clinical Research Unit, London Health Sciences Centre, Ontario, Canada
5. Faculty of Medicine & Health Sciences, University of Nottingham, United Kingdom

Email addresses

Helen MacLaughlin: helen.maclaughlin@nhs.net
Rochelle M Blacklock: Rochelle.blacklock@nhs.net
Kelly Wright: kelly.wright6@nhs.net
Gerda Pot: gerda.pot@kcl.ac.uk
Satish Jayawardene: satish.jayawardene@nhs.net
Christopher W McIntyre: cmcint48@uwo.ca
Iain C Macdougall: iain.macdougall@nhs.net
Nicholas M Selby: Nicholas.Selby@nottingham.ac.uk

Corresponding Author
Dr Helen MacLaughlin
Department of Nutrition and Dietetics
King's College Hospital
Denmark Hill SE5 9RS
United Kingdom
Telephone: +44 20 3299 6250

Word count: 3844

For peer review only - http://bmjopen.bmj.com/site/about/guidelines.xhtml

Abstract

Objectives: To test the methodology of recruitment, retention, and data completeness in a prospective cohort recruited after a hospitalised episode of acute kidney injury (AKI), to inform a future prospective cohort study examining the effect of obesity on AKI outcomes

Design: Feasibility study

Setting: Single centre, multi-site UK tertiary hospital

Participants: 101 participants (67M; 34F) with a median age of 64 (IQR 53-73) years, with and without obesity, recruited within 3 months of a hospitalised episode of AKI.

Outcome measures: Feasibility outcomes were recruitment (>15% meeting inclusion criteria recruited), participant retention at 6 and 12 months (≥80%), and completeness of data collection. Exploratory measures included recovery from AKI (regaining >75% of pre-AKI estimated glomerular filtration rate (eGFR)) at 6 months, development or progression of chronic kidney disease (CKD) (kidney function decrease of ≥25% + rise in CKD category) at 12 months, and associations with poorer kidney outcomes.

Results: 41% of eligible patients consented to take part, exceeding the target recruitment uptake rate of 15%. Retention was 86% at 6 months and 78% at 12 months; 10 patients died and 3 commenced dialysis during the study. Data were 90-100% complete. Median BMI was 27.9kg/m^2 (range 18.1-54.3kg/m^2). 50% of the cohort had stage 3 AKI, and 49% had pre-existing CKD. 46% of the cohort met the AKI recovery definition at 6 months. At 12 months, 20 patients developed CKD (39%), and CKD progression occurred in 11 patients (22%). Post AKI Interleukin-6 and cystatin C were associated with 12-month decline in eGFR.

Conclusions: Feasibility to conduct a long -term observational study addressing AKI outcomes associated with obesity was demonstrated. A fully powered prospective cohort study to examine the relationships between obesity and outcomes of AKI is warranted.

Strengths and Limitations:
A prospective cohort study was conducted in a generalizable cohort of patient experiencing acute kidney injury, with follow up at 6 and 12 months.
Both creatinine and cystatin-C were measured during follow up to estimate kidney function.
Feasibility outcomes were established prior to study commencement.
Obesity may increase the bias in kidney function prediction equations, which is a limitation of this study.
As a feasibility study, the study is not powered to determine the effect of obesity on outcomes after acute kidney injury.

For peer review only - http://bmjopen.bmj.com/site/about/guidelines.xhtml

Introduction

AKI is defined as a sudden reduction in urine output or rise in serum creatinine [1]. Standardisation of the definition of AKI [2] has enabled the severity, risk factors and mortality associated with AKI to be studied. Hospitals in the National Health Service (NHS) in England and Wales identify episodes of AKI using a standardised algorithm based on serial serum creatinine measures [3]. Around 60% of AKI episodes are stage 1, the least severe, with 20% stage 3, the most severe, and overall in-hospital mortality with AKI is high [4]. Full recovery from AKI was previously thought to be associated with no future disease risk, yet data have emerged from experimental animal studies and human observational cohorts demonstrating an increased likelihood of permanent kidney damage, with an almost 9 times greater risk of later development of chronic kidney disease after AKI, compared to those without AKI [5-9].

Previous studies addressing factors influencing post AKI recovery of kidney function and the future development of consequential chronic kidney disease (CKD) have generally been retrospective and utilised administrative datasets [9]. A recent meta analysis and subsequent large retrospective population cohort study identified pre-existing CKD as a significant modifier of outcomes post AKI, indicating that future studies should classify patients by not only AKI severity, but also by pre-AKI kidney function [10, 11]. In a prospective, matched cohort study, non-recovery of premorbid kidney function at 90-days post AKI was identified as a risk factor for longer term development or progression of CKD, when compared to hospitalised patients without AKI [8].

Observational studies suggest that obesity is an independent risk factor for the development and progression of CKD [12, 13] even after adjustment for the related risk factors hypertension and diabetes [14-16]. Obesity is also associated with an increased risk and greater severity of AKI [17-19]. However, the effect of obesity on kidney outcomes after AKI is not known. Several

For peer review only

For peer review only - http://bmjopen.bmj.com/site/about/guidelines.xhtml

mechanisms have been described by which obesity may carry an increased risk for longer-term kidney damage after AKI. Inflammation and increased intra-abdominal pressure, which may increase AKI risk in obesity, coupled with known obesity-related kidney damage due to glomerulopathy and hyperfiltration [20], may hamper recovery of kidney function or exacerbate existing damage and contribute to an increased risk of CKD development or progression in patients with obesity experiencing AKI. These observations lead to a plausible hypothesis that obesity may influence the risk of future CKD development or progression after AKI.

There are also some methodological challenges in the longitudinal study of renal function in patients with obesity. Interpreting renal recovery with obesity may be complex, as the 4 variable Modification of Diet in Renal Disease study equation [21] in widespread use may underestimate measured glomerular filtration rate (GFR) [22]. Two recent observational studies suggest that the CKD Epidemiology Collaboration (CKD-EPI) equations [23, 24] give closer estimates of measured GFR with obesity [25, 26]. The CKD-EPI creatinine equation has been shown to perform best in a group of patients including those with established CKD [26], whilst the addition of cystatin C to the CKD-EPI equation was the closest predictor of measured GFR in obesity with normal kidney function [25, 26]. In addition, there may be changes in body composition in the period of recovery after an episode of AKI that could also potentially affect estimating equations based on serum creatinine concentration. Prospective studies are required to adequately address these challenges.

Therefore, prior to future, large cohort studies to establish the effect of obesity on long term outcomes after an episode of AKI, we performed a feasibility study to test the proposed study design and establish recruitment, retention and data collection rates.

For peer review only - http://bmjopen.bmj.com/site/about/guidelines.xhtml

For peer review only

Materials and Methods

A prospective cohort study was conducted from June 2015 to May 2017 in a large, multi-site, tertiary referral university hospital in London, UK, providing all major medical and surgical specialties, including a nephrology tertiary referral service. The study was primarily designed to examine the feasibility of recruiting and retaining patients in a cohort study exploring the relationship between obesity and the development or progression of CKD after an episode of AKI. The National Research Ethics Service - London Bloomsbury Committee approved the study (15/LO/0907), and all patients provided written informed consent. The aim was to recruit a sample size of 100 patients over 26 weeks. It was anticipated that at least 30% patients would be obese, as obesity accounts for at least 1 in 8 hospital admissions in women in the UK [27], providing an adequate sample of obese participants in the total sample. This was predicted to be adequate to calculate the rates of recruitment and retention and ease of data collection adequately and efficiently, yet in a large enough sample population to assess feasibility for the full cohort study.

For peer review only

Eligible participants were patients aged 18-85 years who had experienced an AKI at any point during hospitalisation, who also had at least one previous serum creatinine measurement available within the previous 12 months. Baseline kidney function was determined from the most recent stable serum creatinine value available within the last 12 months, using the CKD-EPI creatinine equation [23]. Potential participants were identified by screening reports from an electronic AKI detection system using the NHS algorithm developed with the KDIGO definition of AKI [28], or identified through the tertiary nephrology referral service, or through manual searches of daily serum creatinine values, prior to the implementation of the electronic AKI detection system. Exclusion criteria were pre-AKI kidney function less than 20ml/min/1.73m^2 using the CKD-EPI creatinine formula [23], inability to provide informed consent, receiving palliative care, and

For peer review only - http://bmjopen.bmj.com/site/about/guidelines.xhtml

highly likelihood of death during hospitalisation. A member of the research team confirmed each episode of AKI, and excluded progressive CKD cases without AKI.

Patients were recruited and consented either during or just after their hospital admission with AKI. The baseline study visit was planned to occur within 4 weeks of hospital discharge, and included recording of pre-AKI kidney function, diabetes status, smoking status, medications, age, gender, ethnicity, stage and cause of AKI (if known), measurement of height, weight, waist circumference, and collection of blood and urine samples. Further study visits were completed at 3, 6, and 12 months post AKI onset, and measurements and sample collections were completed according to the schedule in Table 1.

Table 1: Schedule of data collection

Data Collected	Time after AKI	< 4 weeks	3 months	6 months	12 months
Height		•			
Serum leptin, adiponectin		•			
Weight, waist circumference		•			•
Serum IL-6, C-reactive protein		•			•
Serum creatinine, cystatin C		•	•	•	•
Urinary protein to creatinine ratio		•	•	•	•
Additional plasma, serum and urine		•	•	•	•

Using a wall-mounted stadiometer, the height was measured to the nearest 1 cm without the patients wearing shoes. With the patient wearing light clothing and no shoes, weight was measured to the nearest 0.1 kg on a calibrated electronic scale. Waist circumference was measured to the nearest 0.1 cm at the level of the umbilicus with a calibrated plastic tape measure. Obesity was defined as a body mass index (BMI = weight in kilograms/height in meters squared) ≥ 30 kg/m^2 and categories of BMI were described according to the World Health Organisation

For peer review only

For peer review only - http://bmjopen.bmj.com/site/about/guidelines.xhtml

(WHO) classification system for obesity [29]. Conicity index was calculated as waist circumference (m)/0.109 x √(weight (kg)/height (m)). Patients who commenced dialysis during the study follow up period were coded as having progressive CKD, and no further measures of serum creatinine were undertaken.

Pre-specified success criteria for the feasibility outcomes of the study were: (1) at least 15% of those invited to join the study consent to take part; and (2) at least 80% retention at 6 and 12 months. Secondary outcomes included data collection rates, and prevalence of AKI across BMI categories. Associations between baseline BMI, waist circumference and conicity index and markers of adiposity, inflammation, proteinuria, and the change in CKD-EPI creatinine eGFR from pre-AKI to 12 months post AKI were examined using Spearman rank correlation, in patients with, and without pre-existing CKD. Exploratory outcomes included the degree of recovery from AKI up to six months post AKI by baseline body mass index (BMI), the prevalence of development or progression of CKD 12 months post AKI, and a comparison of the CKD-EPI equation for creatinine alone and combined with cystatin-C [24]. Recovery of kidney function was defined as a percentage of the pre-AKI baseline eGFR using the CKD-EPI creatinine estimating equation [23]. Full recovery was >75%, partial recovery 50-75%, and non-recovery if requiring dialysis or <50% of pre-AKI eGFR was retained at 6 months. At 12 months, progression was defined using the KDIGO criteria of a 25% reduction in CKD-EPI creatinine eGFR together with a decline of at least 1 category of CKD staging [2], OR commencement of renal replacement therapy. Risk ratios for recovery at 6 months and development or progression of CKD at 12 months in obesity compared to normal BMI were calculated separately for those with and without pre-existing CKD.

Patient and Public Involvement

For peer review only - http://bmjopen.bmj.com/site/about/guidelines.xhtml

Patients and public representatives were consulted on the methods of recruitment and the burden of study follow up visits. As a result of the consultation the recruitment period was extended post discharge to reduce the burden of decision making at the time of acute illness. Follow up study visits were extended to all hospital sites as a result of the consultation. Patient representatives reviewed and refined the patient information sheet and lay summary of the study design. All patients were informed of the study findings by letter and were invited to attend 1 of 2 group sessions to give feedback on how the study was conducted and developed a summary of the study findings.

Results

Participants

Recruitment took place from June 2015 - May 2016. During the recruitment period 706 patients were identified as having an episode of AKI, with 242 patients eligible who met the inclusion criteria for the study (Figure 1). Major reasons for ineligibility were age, death in hospital or receiving palliative care, inability to attend study visits due to distance from hospital to usual home address (international or patients from out of area referred into specialist tertiary services), and no pre-AKI serum creatinine value within the previous 12 months. Of the 242 eligible patients, 232 patients were invited to participate in the study by the time the study reached full recruitment. 101 patients consented to participate in the study, one withdrew prior to baseline and one did not attend the baseline assessment and died shortly after, leaving 99 patients in study.

Baseline characteristics for the entire cohort, and by BMI category, are displayed in Table 1. 40% of the patients with AKI were obese, 29% were overweight, and 31% had a normal BMI. Median BMI was 27.9 kg/m^2 (range 18.1 kg/m^2 - 54.3 kg/m^2). Two patients with a BMI between 18.1 - 18.5 kg/m^2 were incorporated into the normal BMI group. 28% of patients had AKI stage 1, 20% had AKI stage 2 and 52% had AKI stage 3 - with 45% of these requiring renal

For peer review only - http://bmjopen.bmj.com/site/about/guidelines.xhtml

replacement therapy during their hospital admission. Median CKD-EPI creatinine eGFR was 60ml/min/1.73m^2, and 48% of the cohort had pre-existing CKD stage 3 or higher prior to entry into the study. 70 patients had a predominantly likely pre-renal cause for their AKI, 11 patients had a primarily obstructive cause, 12 patients had AKI attributed to the kidenys, and in 6 patients the AKI was deemed to have been multifactorial.

Recruitment and retention

Screening and recruitment for a target of 100 patients was completed in 12 months; 6 months longer than the planned recruitment time. Electronic identification of AKI was not available for the first 9 months of recruitment so manual searches of electronic data were conducted. Over the 12 months of recruitment, on average 59 patients were screened each month, 20 were eligible for the study and 8.4 patients per month consented to participate. Overall, 41% of eligible patients approached to join the study consented to take part, exceeding the target recruitment uptake rate of 15%.

Table 1: Baseline characteristics for (median interquartile range) of patients with acute kidney injury in a hospital population. Data are presented as median (interquartile range) or counts.

	BMI 18-24.9 kg/m²	BMI 25-29.9 kg/m²	BMI ≥ 30 kg/m²
Participants	31	28	40
Age (years)	69 (50, 74)	64 (60, 71)	59 (53, 73)
Sex	21M; 10F	19M; 9F	27M; 13F
Ethnicity	22 White<br>9 Black/Asian/Other	18 White<br>10 Black/Asian/Other	22 White<br>18 Black/Asian/Other
Weight (kg)	60.6 (55.7, 67)	80.6 (73.1, 83.9)	103.2 (94.4, 114.4)
BMI (kg/m²)	21.3 (19.8, 23.5)	26.7 (25.6, 28.5)	35.7 (31.9, 38.8)
Waist circumference (cm)	88 (82.5, 92.8)	103.6 (98.8, 109)	123 (115.1, 131.5)
Conicity Index	1.32 (1.27, 1.37)	1.42 (1.35, 1.46)	1.44 (1.35, 1.49)
Existing CKD (n)	14	12	22
Hypertension (n)	16	20	30
Diabetes (n)	4	15	17
CKD-EPI creatinine	61 (43, 78)	61 (48, 77)	55 (46, 86)

For peer review only - http://bmjopen.bmj.com/site/about/guidelines.xhtml

eGFR (ml/min/1.73m²)			
AKI stage			
1	9 (32%)	9 (32%)	10 (36%)
2	8 (40%)	2 (10%)	10 (50%)
3	14 (27%)	17(33%)	20 (40%)
Required dialysis	8/14	5/17	10/20

AKI – acute kidney injury; BMI – body mass index; CKD – chronic kidney disease; CKD-EPI – CKD Epidemiology Collaboration; eGFR – estimated glomerular filtration rate

By 6 months, a total of 3 patients had died, and 8 patients withdrew, including 1 who was too unwell to provide ongoing consent. This left 88 patients in the study, including 2 requiring dialysis (88/101, 87%), although 6 did not have a study visit at 6 months (3 did not attend, 3 declined the visit but remained in the study). By 12 months, 10 patients had died, 12 had withdrawn, and 79 remained in the study, including 3 requiring dialysis, giving a final retention rate of 78% (79/101) of consenting patients in the study at 12 months. 86% of patients still living 12 months post AKI, attended their final study visit.

Data were 100% complete for height, weight, reason for admission, diabetes and hypertension status, blood sampling and AKI staging including identifying the likely cause and classification as pre-renal, renal or post-renal. Urine sampling was 96% complete and waist circumference was measured in 90% of the sample. The median time to the baseline study visit from the date of the peak creatinine was 44 (IQR 32-58) days. Baseline data collection was planned to be completed 1-month post hospital discharge after AKI. However, many patients were either still hospitalised, or not well enough or ready to consider providing informed consent at this point, so the protocol was amended to enable the baseline visit to be completed within 3 months of the AKI episode. Median time to 6 and 12-month visits were 186 (180-193) days and 367 (361-373) days, respectively.

Kidney function and BMI

For peer review only

For peer review only - http://bmjopen.bmj.com/site/about/guidelines.xhtml

Estimated kidney function at all study time points is displayed in Figure 2, using the CKD-EPI creatinine formula, from pre-AKI to 12 months after AKI (left) and with the CKD-EPI creatinine + cystatin-C formula from baseline to 12 months (right). There is a clear decline in eGFR which persists at 12 months post AKI, particularly in the non-CKD group. With obesity, there is greater undulation in the eGFR recovery pattern compared to the other BMI groups.

The incidence rates for eGFR recovery at 6 months and development or progression of CKD at 12 months for patients are displayed in table 3. At 6 months, 46 patients had recovered >75% of baseline eGFR, 31 had partial recovery of eGFR (50-75%), 7 had not recovered (<50% baseline eGFR, or dialysis), 3 patients had died, and 14 had no data available. In those with normal kidney function pre-AKI, the relative risk for recovery of baseline kidney function at 6 months with obesity, was not significant (relative risk 0.75; 95% CI 0.3-1.7) compared to normal BMI. In patients with pre-existing CKD, there was also no difference in likelihood of recovery to baseline eGFR between obesity and normal BMI (relative risk 1.3; 95% CI 0.8-2.4).

Table 3: Kidney function recovery at 6 months and chronic kidney disease incidence and progression 12 months post acute kidney injury

	BMI 18-24.9 kg/m²		BMI 25-29.9 kg/m²		BMI ≥ 30 kg/m²	
**Recovery > 75% eGFR at 6 months**						
No CKD	47.1%	8/17	69.2%	9/13	35.3%	6/17
Pre-existing CKD	47.1%	8/13	50%	5/10	71.4%	10/14
**Progression >25% decline and decrease in CKD stage at 12 months**						
No CKD	69.2%	9/13	41.7%	5/12	37.5%	6/16
Pre-existing CKD	27.3%	3/11	27.3%	3/11	31.3%	5/16

BMI – body mass index; CKD – chronic kidney disease, eGFR – estimated glomerular filtration rate

At 12 months, 10 patients had died, 31 patients had progressive or incident CKD, including 2 requiring renal replacement therapy. 48 patients had recovered pre-AKI kidney function, and 12 had withdrawn from the study. In patients with pre-existing CKD, progression occurred in 11 patients (22%). In those with normal kidney function, 20 patients developed CKD after AKI (39%). Obesity did not significantly impact on the relative risk of CKD development (relative

For peer review only - http://bmjopen.bmj.com/site/about/guidelines.xhtml

risk = 0.5; 95% CI 0.3-1.1) or progression (relative risk = 1.15; 95% CI 0.3-3.8), compared to normal BMI.

Median values for clinical parameters and markers of inflammation at baseline and 12 months are displayed in Table 4, stratified by BMI category and pre-AKI CKD status. Generally, there were trends for increasing weight and decreasing inflammation over the 12 months following AKI in all groups. Proteinuria tended to decrease over time in the non-CKD group, and increase in the CKD group.

Table 4: Clinical parameters and markers of inflammation at 1-3 months and 12 months after acute kidney injury (median and IQR)

	BMI 18-24.9 kg/m²		BMI 25-29.9 kg/m²		BMI ≥ 30 kg/m²	
Post AKI	1-3M	12M	1-3M	12M	1-3M	12M
**No CKD**						
weight (kg)	62.1 (58.1, 66.7)	67.5 (59.6, 74.9)	83.1 (77.1, 85.6)	92.0 (76.1, 97.3)	110.9 (97.7, 117.7)	115.0 (110.8, 124.0)
hs-CRP (mg/L)	9.3 (4.1, 29.6)	3.2 (1.35, 13.3)	7.65 (3.05, 10.95)	5.1 (2.7, 21.6)	10.6 (6.2, 17.8)	9.0 (3.9, 16.25)
IL-6 (ng/L)	5.14, (3.2, 11.62)	1.21 (1.17, 7.44)	7.89 (3.92, 15.54)	11.42 (8.52, 26.74)	10.75 (5.04, 18.47)	8.63 (3.72, 9.24)
PCR (mg/mmol)	20.0 (8.3, 41.9)	7.6 (6.1, 16.2)	11.7 (6.3, 64.8)	13.5 (5.0, 55.4)	20.5 (7.1, 63.9)	10.3 (6.8, 20.4)
**Pre-existing CKD**						
weight (kg)	57.9 (54.6, 67.0)	63.2 (58.9, 73.0)	75.6 (72.5, 81.1)	77.8 (73.8, 84.4)	96.4 (90.3, 107.6)	98.9 (83.0, 116.0)
hs-CRP (mg/L)	3.4 (1.4, 9.6)	2.4 (1.5, 16.4)	6.05 (4.4, 20.15)	4.1 (0.65, 7.45)	8.15 (3.7, 19.2)	7.0 (2.8, 14.8)
IL-6 (ng/L)	6.12 (4.51, 10.23)	4.63 (3.08, 6.62)	10.55 (7.55, 18.6)	7.26 (5.28, 10.0)	6.36 (4.22, 10.02)	5.64 (3.62, 11.0)
PCR (mg/mmol)	21.5 (11.3, 36.2)	24.2 (7.5, 86.6)	28.9 (11.2, 157.4)	35.8 (17.7, 201.0)	26.3 (7.8, 75.3)	33.6 (6.0, 62.8)

BMI – body mass index; CKD – chronic kidney disease; hs-CRP – high sensitivity c-reactive protein; IL-6 – interleukin-6; PCR – urinary protein to creatinine ratio

For peer review only - http://bmjopen.bmj.com/site/about/guidelines.xhtml

Associations between baseline BMI and 12 month change in CKD-EPI creatinine eGFR and potential markers of risk of CKD progression are displayed in Table 5. Baseline IL-6 was inversely associated with change in eGFR over 12 months in the non CKD group, and in the pre-existing CKD group and cystatin C inversely associated with CKD progression in the non CKD group only. No other risk markers, including BMI, were significantly associated with eGFR change. Leptin, waist circumference and conicity index were strongly associated with BMI, but not with change in eGFR.

Table 5: Spearman rank correlations between body mass index or 12 month change in eGFR with baseline measures of adiposity, inflammation and kidney damage

	adiponectin	leptin	IL-6	hs-CRP	PCR	Waist	conicity index	BMI	cystatin c
**No CKD**									
BMI	-0.087	**0.774**	0.262	0.164	-0.093	**0.948**	**0.599**	1	-0.01
p	0.5	<0.001	0.06	0.3	0.5	<0.001	<0.001	-	0.9
eGFR change	0.07	-0.083	**-0.361**	-0.152	-0.138	0.065	-0.026	0.031	**-0.44**
p	0.7	0.6	0.02	0.4	0.4	0.7	0.8	0.8	<0.01
**Pre-existing CKD**									
BMI	**-0.310**	**0.666**	-0.062	0.22	0.015	**0.921**	**0.396**	1	-0.1
p	0.03	<0.001	0.7	0.1	0.9	<0.001	<0.01	-	0.5
eGFR change	-0.18	0.281	**-0.410**	-0.021	-0.21	0.186	0.098	0.215	-0.05
p	0.3	0.1	0.02	0.9	0.2	0.3	0.6	0.2	0.8

BMI – body mass index; CKD – chronic kidney disease; eGFR – estimated glomerular filtration rate; hs-CRP – high sensitivity c-reactive protein; IL-6 – interleukin-6; PCR – urinary protein to creatinine ratio

Bland-Altman plots of the mean versus difference for CKD-EPI creatinine and creatinine + cystatin-C eGFR equations, by BMI category, are presented in Figure 3. CKD-EPI creatinine eGFR was consistently higher than CKD-EPI creatinine + cystatin-C eGFR across all categories of BMI, and this effect was more marked at higher eGFR levels.

For peer review only - http://bmjopen.bmj.com/site/about/guidelines.xhtml

For peer review only

Discussion

Feasibility

We have demonstrated that it is possible to recruit and retain patients in a study of longer term outcomes associated with obesity after hospitalisation with AKI. Recruitment was well above the target, indicating that patients are willing to participate in studies monitoring kidney function after an episode of AKI. Learning form the workload of this study, we expect that one full time researcher would be required to recruit at the target rate of 5 patients per week, and complete the study data collection visits.

Retention was just below target, due to patient withdrawal and the competing risk of death. When determining the sample size for a longer term study on obesity associated outcomes after AKI it will be important to account for loss of follow up due to the competing risk of death, as 1 and 5 year mortality post AKI can be >40% and >60% respectively, in studies utilising healthcare data [30].

Timing of data collection and selection of study variables

The initial study visit 1 month post the peak creatinine was demonstrated not to be feasible, as only one quarter of patients were able to complete the baseline visit within this time frame. As in other prospective studies, performing the baseline study visit 3 months post the AKI episode is recommended [31, 32].

In this feasibility study, data were collected with a view to determining the optimal markers to select for measurement in larger prospective observational studies, and to formulate hypotheses for mechanisms of obesity associated outcomes after AKI. Waist circumference and conicity index correlated highly with BMI, however neither measure discriminated differently to BMI in

For peer review only - http://bmjopen.bmj.com/site/about/guidelines.xhtml

associations with change in eGFR, adipose tissue hormones, proteinuria or markers of inflammation. Single measures of hormonal markers of adiposity post AKI, such as leptin and adiponectin, may not provide any additional mechanistic understanding over BMI alone. Together, these results indicate that BMI is an adequate classifier of obesity in this population. IL-6 and cystatin-C, as markers of inflammation and kidney damage respectively, are likely to provide insight into prediction of risk for CKD outcomes after AKI, as our feasibility data suggest that IL-6 is a more sensitive than hs-CRP.

Measurement of eGFR in obesity

The utility of prediction equations for eGFR in obesity remains unclear, particularly when body size or body composition is in flux [25, 33, 34]. The CKD-EPI creatinine + cystatin-C equation provided the closest estimate to measured GFR in obesity pre and post bariatric surgery [25], but has not been studied extensively post AKI. We chose to examine it as a potential measure of kidney function in this feasibility study. There appeared to be more undulation in eGFR with both equations in patients with obesity, regardless of underlying level of kidney function. This may be attributed to the known reduced accuracy of eGFR predictions equations with obesity [26], and highlights the importance of using multiple measures of eGFR over time when examining long term change in kidney function. Bland Altman plots did not indicate any difference in the bias between the 2 methods across categories of BMI but did suggest that at higher eGFR levels the estimating equation using cystatin generated lower results than the creatinine only equation. Additionally, there was no association between baseline BMI and cystatin C in this cohort, and together these may indicate that cystatin C was not influenced by obesity in this study. Further studies comparing different estimating equations to measured GFR in post AKI populations are required to determine which methods are valid.

For peer review only - http://bmjopen.bmj.com/site/about/guidelines.xhtml

For peer review only

At all time points post AKI, eGFR was higher using the CKD-EPI creatinine equation, than with the creatinine+cystatin-C equation. Other studies have also found differences in creatinine and cystatin-C based equations in specific populations. In an elderly community population, smoking, age, BMI, inflammation (measured by C-reactive protein) and glucocorticoid were associated with higher creatinine based eGFR and lower cystatin-C based estimates of GFR [35]. The authors suggested that these differences could be due to a reduction in muscle mass and inflammation, respectively [35]. These non-GFR determinants of creatinine and cystatin-C are both also likely evident after AKI, and may explain the lower eGFRs observed in the current study with the inclusion of cystatin-C. Cystatin-C correlated with indices of adiposity, although in a previous study the difference between measured and cystatin–C based eGFR did not correlate with body fat measured by DEXA in young males with normal kidney function [36]. In that study, the CKD-EPI creatinine + cystatin-C equation performed best for both high lean mass and high fat mass groups, although the creatinine based equation had greater accuracy for the normal BMI group. Together, these findings suggest that both body composition and inflammation are potential confounding factors for interpretation of estimates of kidney function post AKI, due to their non-GFR influences on creatinine and cystatin-C. Studies examining CKD-EPI creatinine + cystatin C eGFR at 90 days post AKI as a risk factor for development or progression of CKD are warranted.

Development and progression of CKD

Almost one-third of patients showed development or progression of CKD 1 year post AKI. Horne and colleagues reported CKD development or progression in 24% of their prospective cohort at 3 years post AKI [8], whilst in those with complete recovery post AKI, development of stage 3 CKD occurred in 15% of patients after median follow up of 2.5 years [5]. 50% of the present cohort experienced stage 3 AKI, whilst most observational cohorts typically include 60-

For peer review only - http://bmjopen.bmj.com/site/about/guidelines.xhtml

For peer review only

70% of patients with stage 1 AKI, [8, 37]. Almost half the cohort had existing CKD and more patients required renal replacement therapy during hospitalisation than in the other UK studies [38], both of which are likely contributors to the high rate of CKD development and progression in the present study.

Raised IL-6 may be a risk factor associated with worsening of eGFR after AKI. One potential contributing factor could be a latent effect of obesity on inflammation, which may influence CKD development after AKI. Whether there is an interaction or "cross-talk" between obesity and markers of inflammation in CKD progression remains to be studied further in a suitably powered study with longer term follow up. This study was not designed to examine if the risk of CKD development or progression is higher with obesity, so the reported lack of difference between obese and non-obese groups could represent type II error. In addition to small sample size, we also recognise the limitation of short-term follow up of 1 year, and it is possible that risks and outcomes associated with obesity will be more pronounced over longer periods of time.

In conclusion, we have demonstrated that it is feasible to conduct a prospective study on the effect of obesity on the long term outcomes after AKI. Our results inform the design of future definitive studies, which should include measures of pre-AKI kidney function, long-term follow up after the acute illness and recovery phase, and careful consideration of the use of creatinine, cystatin-C, or measured GFR to assess kidney function.

a. Contributorship statement: HM, CM, and NS contributed to the conception and design of the study, HM, NS, GP, IM and SJ developed the methodology, HM, SJ, RB & KW collected and analysed the data, HM drafted the work and all authors revised it critically for important intellectual content. All authors approved of the final version submitted and agree to be accountable for all aspects of the work. We would also like to thank Sarah Mackie, Rebecca

For peer review only - http://bmjopen.bmj.com/site/about/guidelines.xhtml

Freeman, Richard Hull, Joble Joseph and Gurminder Khamba for their clinical expertise and assistance in identifying potential participants.

b. Competing interests: None of the authors have any competing interests to declare

c. Funding: This work was supported by a National Institute of Health Research (NIHR)/Higher Education England (HEE) Clinical Lectureship award (CAT CL-2014-05-005). This paper presents independent research funded by the National Institute for Health Research (NIHR) and Health Education England. The views expressed are those of the authors and not necessarily those of the NHS, the NIHR or the Department of Health.

d. data sharing statement: Technical appendix, statistical code and dataset available from corresponding author on request.

References

1. Mehta RL, Kellum JA, Shah SV *et al.* Acute Kidney Injury Network: report of an initiative to improve outcomes in acute kidney injury. Critical care 2007; 11: R31
2. Kidney Disease: Improving Global Outcomes (KDIGO) CKD Work Group. KDIGO 2012 Clinical practice guideline for the evaluation and management of chronic kidney disease. Kidney International Supplements 2013; 3: 1-150
3. NHS England. *Patient Safety Alert. Stage 3 Directive: Standardising the early indentification of Acute Kidney Injury*. Patient Safety Alert 2015 30 January 2015 [cited 2017 10 May]; Available from: https://www.england.nhs.uk/wp-content/uploads/2014/06/psa-aki-alg.pdf.
4. Selby NM, Crowley L, Fluck RJ *et al.* Use of electronic results reporting to diagnose and monitor AKI in hospitalized patients. Clin J Am Soc Nephrol 2012; 7: 533-540
5. Jones J, Holmen J, De Graauw J, Jovanovich A, Thornton S , Chonchol M. Association of complete recovery from acute kidney injury with incident CKD stage 3 and all-cause mortality. Am J Kidney Dis 2012; 60: 402-408
6. Schmitt R, Coca S, Kanbay M, Tinetti ME, Cantley LG , Parikh CR. Recovery of kidney function after acute kidney injury in the elderly: a systematic review and meta-analysis. Am J Kidney Dis 2008; 52: 262-271
7. Venkatachalam MA, Griffin KA, Lan R, Geng H, Saikumar P , Bidani AK. Acute kidney injury: a springboard for progression in chronic kidney disease. Am J Physiol Renal Physiol 2010; 298: F1078-1094
8. Horne KL, Packington R, Monaghan J, Reilly T , Selby NM. Three-year outcomes after acute kidney injury: results of a prospective parallel group cohort study. BMJ Open 2017; 7: e015316

For peer review only - http://bmjopen.bmj.com/site/about/guidelines.xhtml

9. Coca SG, Singanamala S , Parikh CR. Chronic kidney disease after acute kidney injury: a systematic review and meta-analysis. Kidney Int 2012; 81: 442-448
10. Sawhney S, Marks A, Fluck N, Levin A, Prescott G , Black C. Intermediate and Long-term Outcomes of Survivors of Acute Kidney Injury Episodes: A Large Population-Based Cohort Study. Am J Kidney Dis 2016
11. Sawhney S, Mitchell M, Marks A, Fluck N , Black C. Long-term prognosis after acute kidney injury (AKI): what is the role of baseline kidney function and recovery? A systematic review. BMJ Open 2015; 5: e006497
12. Fox CS, Larson MG, Leip EP, Culleton B, Wilson PW , Levy D. Predictors of new-onset kidney disease in a community-based population. Journal of the American Medical Association 2004; 291: 844-850
13. Munkhaugen J, Lydersen S, Wideroe TE , Hallan S. Prehypertension, obesity, and risk of kidney disease: 20-year follow-up of the HUNT I study in Norway. American Journal of Kidney Diseases 2009; 54: 638-646
14. Hsu C, McCulloch CE, Iribarren C, Darbinian J , Go ASW. Body Mass Index and Risk for End-Stage Renal Disease. Ann Intern Med 2006; 144: 21-28
15. Gelber RP, Kurth T, Kausz AT *et al.* Association Between Body Mass Index and CKD in Apparently Healthy Men. American Journal of Kidney Diseases 2005; 46: 871-880
16. Hallan S, de Mutsert R, Carlsen S, Dekker FW, Aasarød K , Holmen J. Obesity, Smoking, and Physical Inactivity as Risk Factors for CKD: Are Men More Vulnerable? American Journal of Kidney Diseases 2006; 47: 396-405
17. Druml W, Metnitz B, Schaden E, Bauer P , Metnitz PG. Impact of body mass on incidence and prognosis of acute kidney injury requiring renal replacement therapy. Intensive Care Med 2010; 36: 1221-1228
18. Soto GJ, Frank AJ, Christiani DC , Gong MN. Body mass index and acute kidney injury in the acute respiratory distress syndrome. Crit Care Med 2012; 40: 2601-2608
19. Shashaty MG, Meyer NJ, Localio AR *et al.* African American race, obesity, and blood product transfusion are risk factors for acute kidney injury in critically ill trauma patients. J Crit Care 2012; 27: 496-504
20. Shen WW, Chen HM, Chen H, Xu F, Li LS , Liu ZH. Obesity-Related Glomerulopathy: Body Mass Index and Proteinuria. Clinical Journal of the American Society of Nephrology 2010; 5: 1401-1409
21. Levey AS, Bosch JP, Lewis JB, Greene T, Rogers N , Roth D. A more accurate method to estimate glomerular filtration rate from serum creatinine: a new prediction equation. Modification of Diet in Renal Disease Study Group. Ann Intern Med 1999; 130: 461-470
22. Delanaye P, Radermecker RP, Rorive M, Depas G , Krzesinski JM. Indexing glomerular filtration rate for body surface area in obese patients is misleading: concept and example. Nephrology Dialysis Transplantation 2005; 20: 2024-2028
23. Levey AS, Stevens LA, Schmid CH *et al.* A new equation to estimate glomerular filtration rate. Ann Intern Med 2009; 150: 604-612
24. Inker LA, Schmid CH, Tighiouart H *et al.* Estimating glomerular filtration rate from serum creatinine and cystatin C. N Engl J Med 2012; 367: 20-29
25. Friedman AN, Moe S, Fadel WF *et al.* Predicting the Glomerular Filtration Rate in Bariatric Surgery Patients. Am J Nephrol 2013; 39: 8-15
26. Lemoine S, Guebre-Egziabher F, Sens F *et al.* Accuracy of GFR Estimation in Obese Patients. Clin J Am Soc Nephrol 2014; 9: 720-727
27. Reeves GK, Balkwill A, Cairns BJ, Green J , Beral V. Hospital admissions in relation to body mass index in UK women: a prospective cohort study. BMC Med 2014; 12: 45
28. Kellum JA, Lameire N, Aspelin P *et al.* Kidney disease: Improving global outcomes (KDIGO) acute kidney injury work group. KDIGO clinical practice guideline for acute kidney injury. Kidney International Supplements 2012; 2: 1-138

For peer review only - http://bmjopen.bmj.com/site/about/guidelines.xhtml

29. World Health Organisation. *BMI classification*. 2006 [cited 2010 11 June]; Available from: http://apps.who.int/bmi/index.jsp?introPage=intro_3.html.
30. Sawhney S, Fluck N, Fraser SD *et al.* KDIGO-based acute kidney injury criteria operate differently in hospitals and the community-findings from a large population cohort. Nephrol Dial Transplant 2016; 31: 922-929
31. Go AS, Parikh CR, Ikizler TA *et al.* The assessment, serial evaluation, and subsequent sequelae of acute kidney injury (ASSESS-AKI) study: design and methods. BMC nephrology 2010; 11: 22
32. Horne KL, Shardlow A, Taal MW , Selby NM. Long Term Outcomes after Acute Kidney Injury: Lessons from the ARID Study. Nephron 2015; 131: 102-106
33. Lieske JC, Collazo-Clavell ML, Sarr MG, Rule AD, Bergstralh EJ , Kumar R. Gastric bypass surgery and measured and estimated GFR in women. Am J Kidney Dis 2014; 64: 663-665
34. Chang AR, Zafar W , Grams ME. Kidney Function in Obesity-Challenges in Indexing and Estimation. Adv Chronic Kidney Dis 2018; 25: 31-40
35. Legrand H, Werner K, Christensson A, Pihlsgard M , Elmstahl S. Prevalence and determinants of differences in cystatin C and creatinine-based estimated glomerular filtration rate in community-dwelling older adults: a cross-sectional study. BMC Nephrol 2017; 18: 350
36. Chew-Harris JS, Florkowski CM, George PM, Elmslie JL , Endre ZH. The relative effects of fat versus muscle mass on cystatin C and estimates of renal function in healthy young men. Annals of clinical biochemistry 2013; 50: 39-46
37. Selby NM, Kolhe NV, McIntyre CW *et al.* Defining the cause of death in hospitalised patients with acute kidney injury. PLoS One 2012; 7: e48580
38. Horne KL, Packington R, Monaghan J, Reilly T, McIntyre CW , Selby NM. The effects of acute kidney injury on long-term renal function and proteinuria in a general hospitalised population. Nephron Clin Pract 2014; 128: 192-200

Figure Legends

Figure 1: Recruitment Flow Diagram

Figure 2: creatinine eGFR (left) and creatinine + cystatin-C eGFR (right) from pre AKI to 12 months post AKI by BMI category with and without pre-existing CKD

Figure 3: Bland Altman plots for eGFR creatinine and eGFR creatinine + cystatin-C for BMI <25 (left), BMI 25-29.9 (middle), and BMI 30+ (right); horizontal lines represent mean difference and 95% limits of agreement

For peer review only - http://bmjopen.bmj.com/site/about/guidelines.xhtml

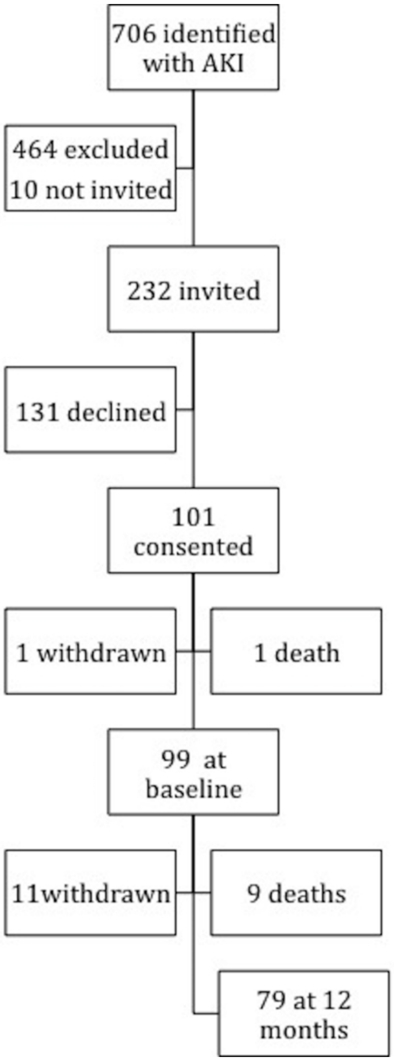

Figure 1: Recruitment Flow Diagram

192x202mm (300 x 300 DPI)

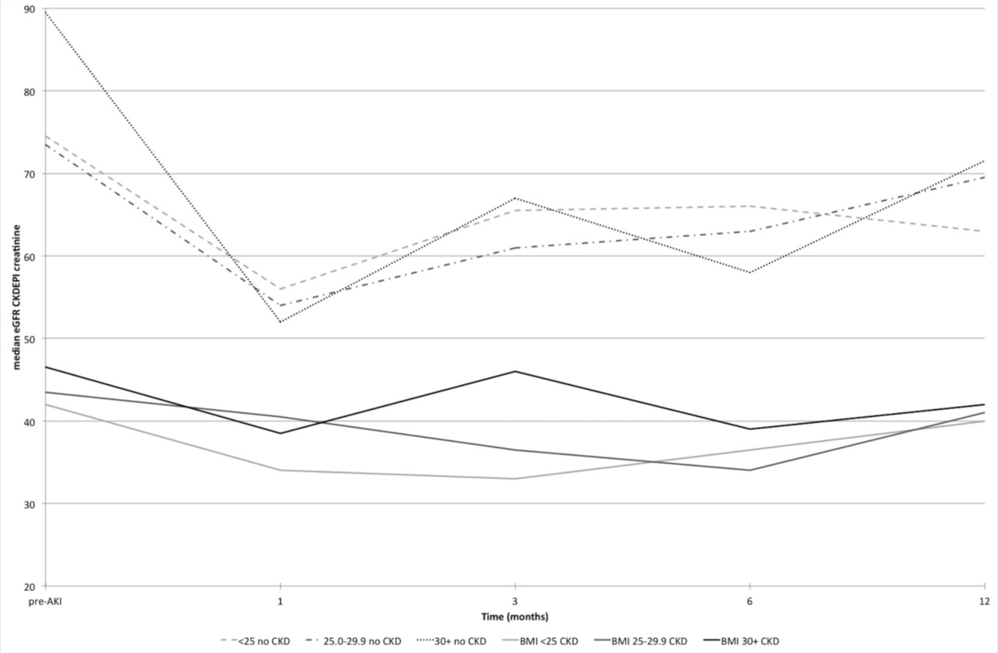

Figure 2: creatinine eGFR (a) and creatinine + cystatin-C eGFR (b) from pre AKI to 12 months post AKI by BMI category with and without pre-existing CKD

573x377mm (300 x 300 DPI)

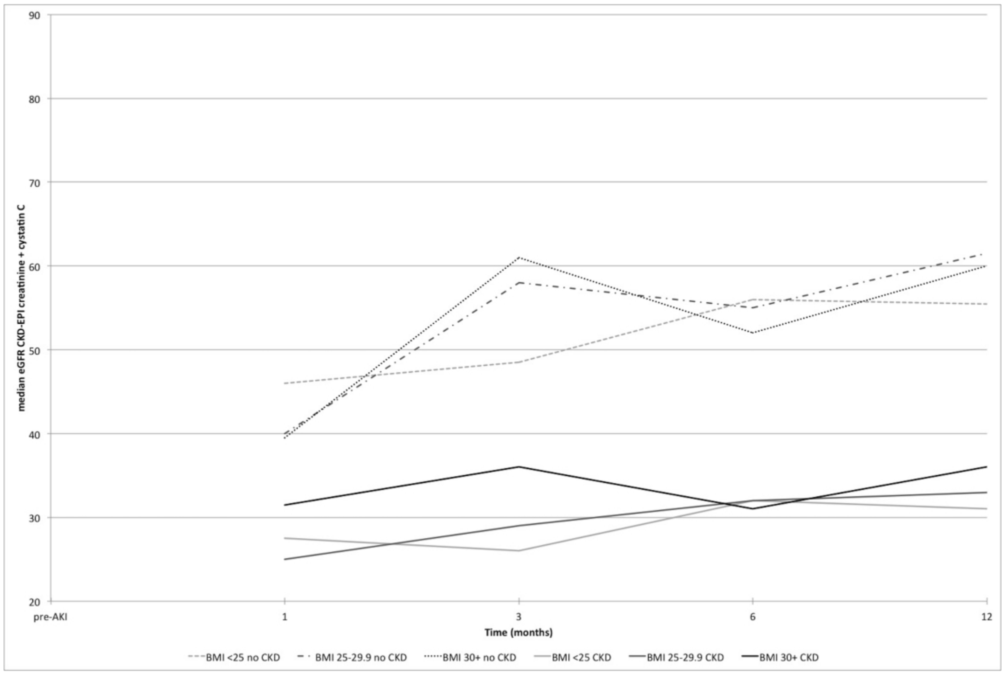

Figure 2: creatinine eGFR (left) and creatinine + cystatin-C eGFR (right) from pre AKI to 12 months post AKI by BMI category with and without pre-existing CKD

563x375mm (300 x 300 DPI)

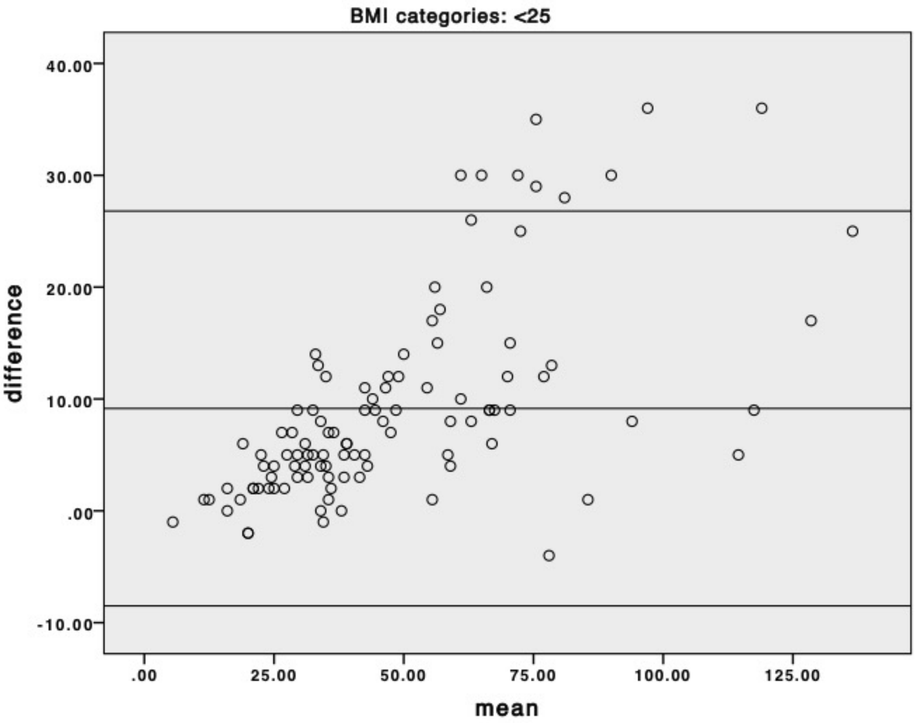

Figure 3: Bland Altman plots for eGFR creatinine and eGFR creatinine + cystatin-C for BMI <25 (a), BMI 25-29.9 (b), and BMI 30+ (c); horizontal lines represent mean difference and 95% limits of agreement

251x201mm (72 x 72 DPI)

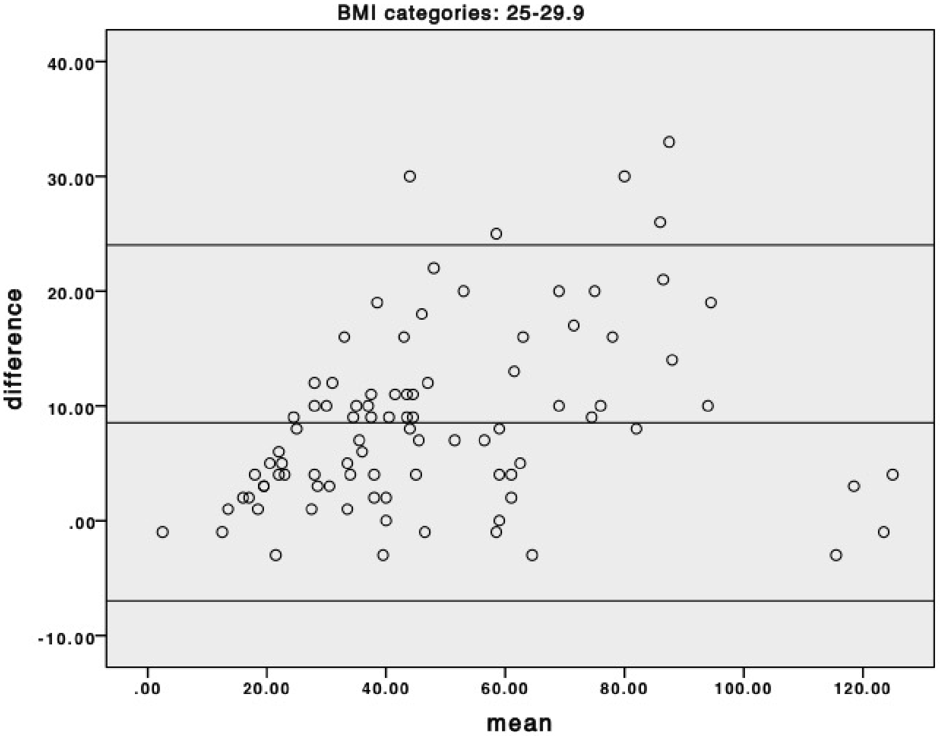

Figure 3: Bland Altman plots for eGFR creatinine and eGFR creatinine + cystatin-C for BMI <25 (a), BMI 25-29.9 (b), and BMI 30+ (c); horizontal lines represent mean difference and 95% limits of agreement

241x190mm (72 x 72 DPI)

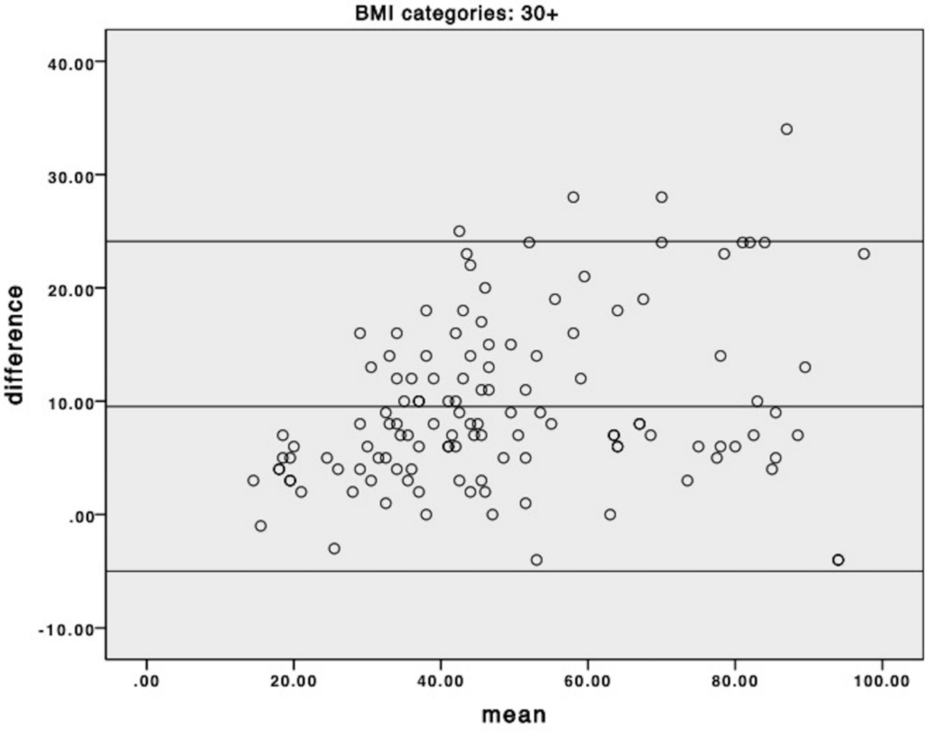

Figure 3: Bland Altman plots for eGFR creatinine and eGFR creatinine + cystatin-C for BMI <25 (a), BMI 25-29.9 (b), and BMI 30+ (c); horizontal lines represent mean difference and 95% limits of agreement

249x194mm (300 x 300 DPI)

STROBE 2007 (v4) Statement—Checklist of items that should be included in reports of *cohort studies*

Section/Topic	Item #	Recommendation	Reported on page #
**Title and abstract**	1	(*a*) Indicate the study's design with a commonly used term in the title or the abstract	1
		(*b*) Provide in the abstract an informative and balanced summary of what was done and what was found	2
**Introduction**			
Background/rationale	2	Explain the scientific background and rationale for the investigation being reported	3-4
Objectives	3	State specific objectives, including any prespecified hypotheses	4
**Methods**			
Study design	4	Present key elements of study design early in the paper	5
Setting	5	Describe the setting, locations, and relevant dates, including periods of recruitment, exposure, follow-up, and data collection	5-6
Participants	6	(*a*) Give the eligibility criteria, and the sources and methods of selection of participants. Describe methods of follow-up	5-6
		(*b*) For matched studies, give matching criteria and number of exposed and unexposed	n/a
Variables	7	Clearly define all outcomes, exposures, predictors, potential confounders, and effect modifiers. Give diagnostic criteria, if applicable	7
Data sources/ measurement	8*	For each variable of interest, give sources of data and details of methods of assessment (measurement). Describe comparability of assessment methods if there is more than one group	6-7
Bias	9	Describe any efforts to address potential sources of bias	n/a
Study size	10	Explain how the study size was arrived at	5
Quantitative variables	11	Explain how quantitative variables were handled in the analyses. If applicable, describe which groupings were chosen and why	6
Statistical methods	12	(*a*) Describe all statistical methods, including those used to control for confounding	7
		(*b*) Describe any methods used to examine subgroups and interactions	7
		(*c*) Explain how missing data were addressed	7
		(*d*) If applicable, explain how loss to follow-up was addressed	
		(*e*) Describe any sensitivity analyses	n/a
**Results**			

For peer review only

Participants	13*	(a) Report numbers of individuals at each stage of study—eg numbers potentially eligible, examined for eligibility, confirmed eligible, included in the study, completing follow-up, and analysed	8
		(b) Give reasons for non-participation at each stage	Fig 1 and 9
		(c) Consider use of a flow diagram	Fig 1
Descriptive data	14*	(a) Give characteristics of study participants (eg demographic, clinical, social) and information on exposures and potential confounders	8-9
		(b) Indicate number of participants with missing data for each variable of interest	10
		(c) Summarise follow-up time (eg, average and total amount)	10
Outcome data	15*	Report numbers of outcome events or summary measures over time	Table 3
Main results	16	(*a*) Give unadjusted estimates and, if applicable, confounder-adjusted estimates and their precision (eg, 95% confidence interval). Make clear which confounders were adjusted for and why they were included	11
		(*b*) Report category boundaries when continuous variables were categorized	11
		(*c*) If relevant, consider translating estimates of relative risk into absolute risk for a meaningful time period	n/a
Other analyses	17	Report other analyses done—eg analyses of subgroups and interactions, and sensitivity analyses	11-12
**Discussion**			
Key results	18	Summarise key results with reference to study objectives	13, 16
**Limitations**			
Interpretation	20	Give a cautious overall interpretation of results considering objectives, limitations, multiplicity of analyses, results from similar studies, and other relevant evidence	14-16
Generalisability	21	Discuss the generalisability (external validity) of the study results	
**Other information**			
Funding	22	Give the source of funding and the role of the funders for the present study and, if applicable, for the original study on which the present article is based	17

For peer review only

*Give information separately for cases and controls in case-control studies and, if applicable, for exposed and unexposed groups in cohort and cross-sectional studies.

Note: An Explanation and Elaboration article discusses each checklist item and gives methodological background and published examples of transparent reporting. The STROBE checklist is best used in conjunction with this article (freely available on the Web sites of PLoS Medicine at http://www.plosmedicine.org/, Annals of Internal Medicine at http://www.annals.org/, and Epidemiology at http://www.epidem.com/). Information on the STROBE Initiative is available at www.strobe-statement.org.

BMJ Open

Obesity and recovery from acute kidney injury (Ob AKI): a prospective cohort feasibility study

Journal:	*BMJ Open*
Manuscript ID	bmjopen-2018-024033.R1
Article Type:	Research
Date Submitted by the Author:	17-Aug-2018
Complete List of Authors:	MacLaughlin, Helen L; King's College Hospital NHS Foundation Trust, Department of Nutrition and Dietetics; King's College London, Department of Nutritional Sciences<br>Blacklock, Rochelle M; King's College Hospital NHS Foundation Trust, Department of Nutrition and Dietetics; King's College London, Department of Nutritional Sciences<br>Wright, Kelly; King's College Hospital NHS Foundation Trust, Department of Renal Medicine<br>Pot, Gerda ; King's College London, Department of Nutritional Sciences<br>Jayawardene, Satish; King's College Hospital NHS Foundation Trust, Department of Renal Medicine<br>McIntyre, CW; University of Western Ontario, Division of Nephrology<br>Macdougall, Iain; Kings College Hospital NHS Foundation Trust, Department of Renal Medicine<br>Selby, Nicholas; University of Nottingham Faculty of Medicine and Health Sciences, Centre for Kidney Research and Innovation; Royal Derby Hospital,
**Primary Subject Heading**:	Epidemiology
Secondary Subject Heading:	Nutrition and metabolism
Keywords:	Acute renal failure < NEPHROLOGY, obesity, Chronic renal failure < NEPHROLOGY, NUTRITION & DIETETICS

SCHOLARONE™
Manuscripts

Obesity and recovery from acute kidney injury (Ob AKI): a prospective cohort feasibility study

Helen L MacLaughlin[1,2], Rochelle M Blacklock[1,2], Kelly Wright[3], Gerda K Pot[2], Satish Jayawardene[3], Christopher W McIntyre[4], Iain C Macdougall[3], Nicholas M Selby[5]

Institutions

1. Department Nutrition and Dietetics, King's College Hospital, London, United Kingdom
2. Department of Nutritional Sciences, Faculty of Life Sciences and Medicine, King's College London, United Kingdom
3. Department of Renal Medicine, King's College Hospital, London, United Kingdom
4. London Kidney Clinical Research Unit, London Health Sciences Centre, Ontario, Canada
5. Faculty of Medicine & Health Sciences, University of Nottingham, United Kingdom

Email addresses

Helen MacLaughlin: helen.maclaughlin@nhs.net
Rochelle M Blacklock: Rochelle.blacklock@nhs.net
Kelly Wright: kelly.wright6@nhs.net
Gerda Pot: gerda.pot@kcl.ac.uk
Satish Jayawardene: satish.jayawardene@nhs.net
Christopher W McIntyre: cmcint48@uwo.ca
Iain C Macdougall: iain.macdougall@nhs.net
Nicholas M Selby: Nicholas.Selby@nottingham.ac.uk

Corresponding Author
Dr Helen MacLaughlin
Department of Nutrition and Dietetics
King's College Hospital
Denmark Hill SE5 9RS
United Kingdom
Telephone: +44 20 3299 6250

Word count: 3844

For peer review only

Abstract

Objectives: To test the methodology of recruitment, retention, and data completeness in a prospective cohort recruited after a hospitalised episode of acute kidney injury (AKI), to inform a future prospective cohort study examining the effect of obesity on AKI outcomes

Design: Feasibility study

Setting: Single centre, multi-site UK tertiary hospital

Participants: 101 participants (67M; 34F) with a median age of 64 (IQR 53-73) years, with and without obesity, recruited within 3 months of a hospitalised episode of AKI.

Outcome measures: Feasibility outcomes were recruitment (>15% meeting inclusion criteria recruited), participant retention at 6 and 12 months (≥80%), and completeness of data collection. Exploratory measures included recovery from AKI (regaining >75% of pre-AKI estimated glomerular filtration rate (eGFR)) at 6 months, development or progression of chronic kidney disease (CKD) (kidney function decrease of ≥25% + rise in CKD category) at 12 months, and associations with poorer kidney outcomes.

Results: 41% of eligible patients consented to take part, exceeding the target recruitment uptake rate of 15%. Retention was 86% at 6 months and 78% at 12 months; 10 patients died and 3 commenced dialysis during the study. Data were 90-100% complete. Median BMI was 27.9kg/m^2 (range 18.1-54.3kg/m^2). 50% of the cohort had stage 3 AKI, and 49% had pre-existing CKD. 46% of the cohort met the AKI recovery definition at 6 months. At 12 months, 20/51 patients developed CKD (39%), and CKD progression occurred in 11/49 patients (22%). Post AKI Interleukin-6 and cystatin C were associated with 12-month decline in eGFR.

Conclusions: Feasibility to conduct a long-term observational study addressing AKI outcomes associated with obesity was demonstrated. A fully powered prospective cohort study to examine the relationships between obesity and outcomes of AKI is warranted.

Strengths and Limitations:
A prospective cohort study was conducted in a generalizable cohort of patient experiencing acute kidney injury, with follow up at 6 and 12 months.
Both creatinine and cystatin-C were measured during follow up to estimate kidney function.
Feasibility outcomes were established prior to study commencement.
Obesity may increase the bias in kidney function prediction equations, which is a limitation of this study.
As a feasibility study, the study is not powered to determine the effect of obesity on outcomes after acute kidney injury.

For peer review only

Introduction

AKI is defined as a sudden reduction in urine output or rise in serum creatinine [1]. Standardisation of the definition of AKI [2] has enabled the severity, risk factors and mortality associated with AKI to be studied. Hospitals in the National Health Service (NHS) in England and Wales identify episodes of AKI using a standardised algorithm based on serial serum creatinine measures [3]. Around 60% of AKI episodes are stage 1, the least severe, with 20% stage 3, the most severe, and overall in-hospital mortality with AKI is high [4]. Full recovery from AKI was previously thought to be associated with no future disease risk, yet data have emerged from experimental animal studies and human observational cohorts demonstrating an increased likelihood of permanent kidney damage, with an almost 9 times greater risk of later development of chronic kidney disease after AKI, compared to those without AKI [5-9].

Previous studies addressing factors influencing post AKI recovery of kidney function and the future development of consequential chronic kidney disease (CKD) have generally been retrospective and utilised administrative datasets [9]. A recent meta analysis and subsequent large retrospective population cohort study identified pre-existing CKD as a significant modifier of outcomes post AKI, indicating that future studies should classify patients by not only AKI severity, but also by pre-AKI kidney function [10, 11]. In a prospective, matched cohort study, non-recovery of premorbid kidney function at 90-days post AKI was identified as a risk factor for longer term development or progression of CKD, when compared to hospitalised patients without AKI [8].

Observational studies suggest that obesity is an independent risk factor for the development and progression of CKD [12, 13] even after adjustment for the related risk factors hypertension and diabetes [14-16]. Obesity is also associated with an increased risk and greater severity of AKI [17-19]. However, the effect of obesity on kidney outcomes after AKI is not known. Several

For peer review only - http://bmjopen.bmj.com/site/about/guidelines.xhtml

For peer review only

mechanisms have been described by which obesity may carry an increased risk for longer-term kidney damage after AKI. Inflammation and increased intra-abdominal pressure, which may increase AKI risk in obesity, coupled with known obesity-related kidney damage due to glomerulopathy and hyperfiltration [20], may hamper recovery of kidney function or exacerbate existing damage and contribute to an increased risk of CKD development or progression in patients with obesity experiencing AKI. These observations lead to a plausible hypothesis that obesity may influence the risk of future CKD development or progression after AKI.

There are also some methodological challenges in the longitudinal study of renal function in patients with obesity. Interpreting renal recovery with obesity may be complex, as the 4 variable Modification of Diet in Renal Disease study equation [21] in widespread use may underestimate measured glomerular filtration rate (GFR) [22]. Two recent observational studies suggest that the CKD Epidemiology Collaboration (CKD-EPI) equations [23, 24] give closer estimates of measured GFR with obesity [25, 26]. The CKD-EPI creatinine equation has been shown to perform best in a group of patients including those with established CKD [26], whilst the addition of cystatin C to the CKD-EPI equation was the closest predictor of measured GFR in obesity with normal kidney function [25, 26]. In addition, there may be changes in body composition in the period of recovery after an episode of AKI that could also potentially affect estimating equations based on serum creatinine concentration. Prospective studies are required to adequately address these challenges.

Therefore, prior to future, large cohort studies to establish the effect of obesity on long term outcomes after an episode of AKI, we performed a feasibility study to test the proposed study design and establish recruitment, retention and data collection rates.

For peer review only

Materials and Methods

A prospective cohort study was conducted from June 2015 to May 2017 in a large, multi-site, tertiary referral university hospital in London, UK, providing all major medical and surgical specialties, including a nephrology tertiary referral service. The study was primarily designed to examine the feasibility of recruiting and retaining patients in a cohort study exploring the relationship between obesity and the development or progression of CKD after an episode of AKI. The National Research Ethics Service - London Bloomsbury Committee approved the study (15/LO/0907), and all patients provided written informed consent. The aim was to recruit a sample size of 100 patients over 26 weeks. It was anticipated that at least 30% patients would be obese, as obesity accounts for at least 1 in 8 hospital admissions in women in the UK [27], providing an adequate sample of obese participants in the total sample. This was predicted to be adequate to calculate the rates of recruitment and retention and ease of data collection adequately and efficiently, yet in a large enough sample population to assess feasibility for the full cohort study.

Eligible participants were patients aged 18-85 years who had experienced an AKI at any point during hospitalisation, who also had at least one previous serum creatinine measurement available within the previous 12 months. Baseline kidney function was determined from the most recent stable serum creatinine value available within the last 12 months, using the CKD-EPI creatinine equation to determine estimated glomerular filtration rate ($eGFR_{creat}$) [23]. Potential participants were identified by screening reports from an electronic AKI detection system using the NHS algorithm developed with the KDIGO definition of AKI [28], or identified through the tertiary nephrology referral service using the full KDIGO definition of AKI, or through manual searches of daily serum creatinine values, prior to the implementation of the electronic AKI detection system. Exclusion criteria were pre-AKI $eGFR_{creat}$ less than 20ml/min/1.73m^2 [23], inability to provide informed consent, receiving palliative care, and highly likelihood of death during

For peer review only - http://bmjopen.bmj.com/site/about/guidelines.xhtml

hospitalisation. A member of the research team confirmed each episode of AKI, and excluded progressive CKD cases without AKI.

Patients were recruited and consented either during or just after their hospital admission with AKI. The baseline study visit was planned to occur within 4 weeks of hospital discharge, and included recording of pre-AKI kidney function, diabetes status, smoking status, medications, age, gender, ethnicity, stage and cause of AKI (if known), measurement of height, weight, waist circumference, and collection of blood and urine samples. Further study visits were completed at 3, 6, and 12 months post AKI onset, and measurements and sample collections were completed according to the schedule in Table 1.

Table 1: Schedule of data collection

Data Collected	Time after AKI	< 4 weeks	3 months	6 months	12 months
Height		•			
Serum leptin, adiponectin		•			
Weight, waist circumference		•			•
Serum IL-6, C-reactive protein		•			•
Serum creatinine, cystatin C		•	•	•	•
Urinary protein to creatinine ratio		•	•	•	•
Additional plasma, serum and urine		•	•	•	•

Using a wall-mounted stadiometer, the height was measured to the nearest 1 cm without the patients wearing shoes. With the patient wearing light clothing and no shoes, weight was measured to the nearest 0.1 kg on a calibrated electronic scale. Waist circumference was measured to the nearest 0.1 cm at the level of the umbilicus with a calibrated plastic tape measure. Obesity was defined as a body mass index (BMI = weight in kilograms/height in meters squared) $\geq$30 kg/m^2 and categories of BMI were described according to the World Health Organisation

For peer review only - http://bmjopen.bmj.com/site/about/guidelines.xhtml

(WHO) classification system for obesity [29]. Conicity index was calculated as waist circumference (m)/0.109 x √(weight (kg)/height (m)). Patients who commenced dialysis during the study follow up period were coded as having progressive CKD, and no further measures of serum creatinine were undertaken.

Pre-specified success criteria for the feasibility outcomes of the study were: (1) at least 15% of those invited to join the study consent to take part; and (2) at least 80% retention at 6 and 12 months. Secondary outcomes included data collection rates, and prevalence of AKI across BMI categories. Associations between baseline BMI, waist circumference and conicity index and markers of adiposity, inflammation, proteinuria, and the change in $eGFR_{creat}$ from pre-AKI to 12 months post AKI were examined using Spearman rank correlation, in patients with, and without pre-existing CKD. Exploratory outcomes included the degree of recovery from AKI up to six months post AKI by baseline body mass index (BMI), the prevalence of development or progression of CKD 12 months post AKI, and a comparison of estimates of kidney function using $eGFR_{creat}$ and the CKD-EPI equation for creatinine combined with cystatin-C ($eGFR_{creat+cysC}$) [24]. Recovery of kidney function was defined as a percentage of the pre-AKI baseline $eGFR_{creat}$ [23]. Full recovery was >75%, partial recovery 50-75%, and non-recovery if requiring dialysis or <50% of pre-AKI $eGFR_{creat}$ was retained at 6 months. At 12 months, progression was defined using the KDIGO criteria of a 25% reduction in $eGFR_{creat}$ together with a decline of at least 1 category of CKD staging [2], OR commencement of renal replacement therapy. Risk ratios for recovery at 6 months and development or progression of CKD at 12 months in obesity compared to normal BMI were calculated separately for those with and without pre-existing CKD.

Patient and Public Involvement

For peer review only - http://bmjopen.bmj.com/site/about/guidelines.xhtml

Patients and public representatives were consulted on the methods of recruitment and the burden of study follow up visits. As a result of the consultation the recruitment period was extended post discharge to reduce the burden of decision making at the time of acute illness. Follow up study visits were extended to all hospital sites as a result of the consultation. Patient representatives reviewed and refined the patient information sheet and lay summary of the study design. All patients were informed of the study findings by letter and were invited to attend 1 of 2 group sessions to give feedback on how the study was conducted and developed a summary of the study findings.

For peer review only

Results

Participants

Recruitment took place from June 2015 - May 2016. During the recruitment period 706 patients were identified as having an episode of AKI, with 242 patients eligible who met the inclusion criteria for the study (Figure 1). Major reasons for ineligibility were age, death in hospital or receiving palliative care, inability to attend study visits due to distance from hospital to usual home address (international or patients from out of area referred into specialist tertiary services), and no pre-AKI serum creatinine value within the previous 12 months. Of the 242 eligible patients, 232 patients were invited to participate in the study by the time the study reached full recruitment. 101 patients consented to participate in the study, one withdrew prior to baseline and one did not attend the baseline assessment and died shortly after, leaving 99 patients in study.

Baseline characteristics for the entire cohort, and by BMI category, are displayed in Table 2. 40% of the patients with AKI were obese, 29% were overweight, and 31% had a normal BMI. Median BMI was 27.9 kg/m^2 (range 18.1 kg/m^2 - 54.3 kg/m^2). Two patients with a BMI between 18.1 - 18.5 kg/m^2 were incorporated into the normal BMI group. 28% of patients had AKI stage 1, 20% had AKI stage 2 and 52% had AKI stage 3 - with 45% of these requiring renal

For peer review only - http://bmjopen.bmj.com/site/about/guidelines.xhtml

replacement therapy during their hospital admission. Median $eGFR_{creat}$ was 60ml/min/1.73m^2, and 48% of the cohort had pre-existing CKD stage 3 or higher prior to entry into the study. 70 patients had a predominantly likely pre-renal cause for their AKI, 11 patients had a primarily obstructive cause, 12 patients had AKI attributed to the kidenys, and in 6 patients the AKI was deemed to have been multifactorial.

Recruitment and retention

Screening and recruitment for a target of 100 patients was completed in 12 months; 6 months longer than the planned recruitment time. Electronic identification of AKI was not available for the first 9 months of recruitment so manual searches of electronic data were conducted. Over the 12 months of recruitment, on average 59 patients were screened each month, 20 were eligible for the study and 8.4 patients per month consented to participate. Overall, 41% of eligible patients approached to join the study consented to take part, exceeding the target recruitment uptake rate of 15%.

For peer review only

Table 2: Baseline characteristics for (median interquartile range) of patients with acute kidney injury in a hospital population. Data are presented as median (interquartile range) or counts.

	BMI 18-24.9 kg/m^2	BMI 25-29.9 kg/m^2	BMI $\geq$ 30 kg/m^2
Participants	31	28	40
Age (years)	69 (50, 74)	64 (60, 71)	59 (53, 73)
Sex	21M; 10F	19M; 9F	27M; 13F
Ethnicity	22 White<br>9 Black/Asian/Other	18 White<br>10 Black/Asian/Other	22 White<br>18 Black/Asian/Other
Weight (kg)	60.6 (55.7, 67)	80.6 (73.1, 83.9)	103.2 (94.4, 114.4)
BMI (kg/m^2)	21.3 (19.8, 23.5)	26.7 (25.6, 28.5)	35.7 (31.9, 38.8)
Waist circumference (cm)	88 (82.5, 92.8)	103.6 (98.8, 109)	123 (115.1, 131.5)
Conicity Index	1.32 (1.27, 1.37)	1.42 (1.35, 1.46)	1.44 (1.35, 1.49)
Existing CKD (n)	14	12	22
Hypertension (n)	16	20	30
	4	15	17

Diabetes (n)			
$eGFR_{creat}$ (ml/min/1.73m^2)	61 (43, 78)	61 (48, 77)	55 (46, 86)
AKI stage			
1	9 (32%)	9 (32%)	10 (36%)
2	8 (40%)	2 (10%)	10 (50%)
3	14 (27%)	17(33%)	20 (40%)
Required dialysis	8/14	5/17	10/20

AKI – acute kidney injury; BMI – body mass index; CKD – chronic kidney disease; CKD-EPI – CKD Epidemiology Collaboration; eGFR – estimated glomerular filtration rate

By 6 months, a total of 3 patients had died, and 8 patients withdrew, including 1 who was too unwell to provide ongoing consent. This left 88 patients in the study, including 2 requiring dialysis (88/101, 87%), although 6 did not have a study visit at 6 months (3 did not attend, 3 declined the visit but remained in the study). By 12 months, 10 patients had died, 12 had withdrawn, and 79 remained in the study, including 3 requiring dialysis, giving a final retention rate of 78% (79/101) of consenting patients in the study at 12 months. 86% of patients still living 12 months post AKI, attended their final study visit.

Data were 100% complete for height, weight, reason for admission, diabetes and hypertension status, blood sampling and AKI staging including identifying the likely cause and classification as pre-renal, renal or post-renal. Urine sampling was 96% complete and waist circumference was measured in 90% of the sample. The median time to the baseline study visit from the date of the peak creatinine was 44 (IQR 32-58) days. Baseline data collection was planned to be completed 1-month post hospital discharge after AKI. However, many patients were either still hospitalised, or not well enough or ready to consider providing informed consent at this point, so the protocol was amended to enable the baseline visit to be completed within 3 months of the AKI episode. Median time to 6 and 12-month visits were 186 (180-193) days and 367 (361-373) days, respectively.

Kidney function and BMI

For peer review only - http://bmjopen.bmj.com/site/about/guidelines.xhtml

Estimated kidney function at all study time points is displayed in Figure 2, using $eGFR_{creat}$, from pre-AKI to 12 months after AKI, and in Figure 3 and with $eGFR_{creat+cysC}$ from baseline to 12 months. There is a clear decline in eGFR, which persists at 12 months post AKI, particularly in the non-CKD group. With obesity, there is greater undulation in the eGFR recovery pattern compared to the other BMI groups.

The incidence rates for eGFR recovery at 6 months and development or progression of CKD at 12 months for patients are displayed in Table 3. At 6 months, 46 patients had recovered >75% of baseline $eGFR_{creat}$, 31 had partial recovery of $eGFR_{creat}$ (50-75%), 7 had not recovered (<50% baseline $eGFR_{creat}$, or dialysis), 3 patients had died, and 14 had no data available. In those with normal kidney function pre-AKI, the relative risk for recovery of baseline kidney function at 6 months with obesity, was not significant (relative risk 0.75; 95% CI 0.3-1.7) compared to normal BMI. In patients with pre-existing CKD, there was also no difference in likelihood of recovery to baseline $eGFR_{creat}$ between obesity and normal BMI (relative risk 1.3; 95% CI 0.8-2.4).

Table 3: Incidence rates for kidney function recovery at 6 months and chronic kidney disease incidence and progression 12 months post acute kidney injury

	BMI 18-24.9 kg/m^2		BMI 25-29.9 kg/m^2		BMI ≥ 30 kg/m^2	
**Recovery > 75% eGFR at 6 months**						
No CKD	47.1%	8/17	69.2%	9/13	35.3%	6/17
Pre-existing CKD	47.1%	8/13	50%	5/10	71.4%	10/14
**Progression >25% decline and decrease in CKD stage at 12 months**						
No CKD	69.2%	9/13	41.7%	5/12	37.5%	6/16
Pre-existing CKD	27.3%	3/11	27.3%	3/11	31.3%	5/16

BMI – body mass index; CKD – chronic kidney disease, eGFR – estimated glomerular filtration rate

At 12 months, 10 patients had died, 31 patients had progressive or incident CKD, including 2 requiring renal replacement therapy. 48 patients had recovered pre-AKI kidney function, and 12 had withdrawn from the study. In patients with pre-existing CKD, progression occurred in 11 patients (22%). In those with normal kidney function, 20 patients developed CKD after AKI (39%). Obesity did not significantly impact on the relative risk of CKD development (relative

For peer review only - http://bmjopen.bmj.com/site/about/guidelines.xhtml

risk = 0.5; 95% CI 0.3-1.1) or progression (relative risk = 1.15; 95% CI 0.3-3.8), compared to normal BMI.

Median values for clinical parameters and markers of inflammation at baseline and 12 months are displayed in Table 4, stratified by BMI category and pre-AKI CKD status. Generally, there were trends for increasing weight and decreasing inflammation over the 12 months following AKI in all groups. Proteinuria tended to decrease over time in the non-CKD group, and increase in the CKD group.

Table 4: Clinical parameters and markers of inflammation at 1-3 months and 12 months after acute kidney injury (median and IQR)

	BMI 18-24.9 kg/m^2		BMI 25-29.9 kg/m^2		BMI ≥ 30 kg/m^2	
Post AKI	1-3M	12M	1-3M	12M	1-3M	12M
**No CKD**						
weight (kg)	62.1 (58.1, 66.7)	67.5 (59.6, 74.9)	83.1 (77.1, 85.6)	92.0 (76.1, 97.3)	110.9 (97.7, 117.7)	115.0 (110.8, 124.0)
hs-CRP (mg/L)	9.3 (4.1, 29.6)	3.2 (1.35, 13.3)	7.65 (3.05, 10.95)	5.1 (2.7, 21.6)	10.6 (6.2, 17.8)	9.0 (3.9, 16.25)
IL-6 (ng/L)	5.14, (3.2, 11.62)	1.21 (1.17, 7.44)	7.89 (3.92, 15.54)	11.42 (8.52, 26.74)	10.75 (5.04, 18.47)	8.63 (3.72, 9.24)
PCR (mg/mmol)	20.0 (8.3, 41.9)	7.6 (6.1, 16.2)	11.7 (6.3, 64.8)	13.5 (5.0, 55.4)	20.5 (7.1, 63.9)	10.3 (6.8, 20.4)
**Pre-existing CKD**						
weight (kg)	57.9 (54.6, 67.0)	63.2 (58.9, 73.0)	75.6 (72.5, 81.1)	77.8 (73.8, 84.4)	96.4 (90.3, 107.6)	98.9 (83.0, 116.0)
hs-CRP (mg/L)	3.4 (1.4, 9.6)	2.4 (1.5, 16.4)	6.05 (4.4, 20.15)	4.1 (0.65, 7.45)	8.15 (3.7, 19.2)	7.0 (2.8, 14.8)
IL-6 (ng/L)	6.12 (4.51, 10.23)	4.63 (3.08, 6.62)	10.55 (7.55, 18.6)	7.26 (5.28, 10.0)	6.36 (4.22, 10.02)	5.64 (3.62, 11.0)
PCR (mg/mmol)	21.5 (11.3, 36.2)	24.2 (7.5, 86.6)	28.9 (11.2, 157.4)	35.8 (17.7, 201.0)	26.3 (7.8, 75.3)	33.6 (6.0, 62.8)

BMI – body mass index; CKD – chronic kidney disease; hs-CRP – high sensitivity c-reactive protein; IL-6 – interleukin-6; PCR – urinary protein to creatinine ratio

For peer review only - http://bmjopen.bmj.com/site/about/guidelines.xhtml

Associations between baseline BMI and 12 month change in $eGFR_{creat}$ and potential markers of risk of CKD progression are displayed in Table 5. Baseline IL-6 was inversely associated with change in $eGFR_{creat}$ over 12 months in the non CKD group, and in the pre-existing CKD group and cystatin C was inversely associated with CKD progression in the non CKD group only. No other risk markers, including BMI, were significantly associated with $eGFR_{creat}$ change. Leptin, waist circumference and conicity index were strongly associated with BMI, but not with change in $eGFR_{creat}$.

Table 5: Spearman rank correlations between body mass index or 12 month change in eGFR with baseline measures of adiposity, inflammation and kidney damage

	adiponectin	leptin	IL-6	hs-CRP	PCR	Waist	conicity index	BMI	cystatin c
**No CKD**									
BMI	-0.087	**0.774**	0.262	0.164	-0.093	**0.948**	**0.599**	1	-0.01
p	0.5	<0.001	0.06	0.3	0.5	<0.001	<0.001	-	0.9
eGFR change	0.07	-0.083	**-0.361**	-0.152	-0.138	0.065	-0.026	0.031	**-0.44**
p	0.7	0.6	0.02	0.4	0.4	0.7	0.8	0.8	<0.01
**Pre-existing CKD**									
BMI	**-0.310**	**0.666**	-0.062	0.22	0.015	**0.921**	**0.396**	1	-0.1
p	0.03	<0.001	0.7	0.1	0.9	<0.001	<0.01	-	0.5
eGFR change	-0.18	0.281	**-0.410**	-0.021	-0.21	0.186	0.098	0.215	-0.05
p	0.3	0.1	0.02	0.9	0.2	0.3	0.6	0.2	0.8

BMI – body mass index; CKD – chronic kidney disease; eGFR – estimated glomerular filtration rate; hs-CRP – high sensitivity c-reactive protein; IL-6 – interleukin-6; PCR – urinary protein to creatinine ratio

Bland-Altman plots of the mean versus difference for $eGFR_{creat}$ and $eGFR_{creat+cysC}$ equations, by BMI category, are presented in Figures 4-6. $eGFR_{creat}$ was consistently higher than $eGFR_{creat+cysC}$ across all categories of BMI, and this effect was more marked at higher eGFR levels.

For peer review only

For peer review only - http://bmjopen.bmj.com/site/about/guidelines.xhtml

Discussion

Feasibility

We have demonstrated that it is possible to recruit and retain patients in a study of longer term outcomes associated with obesity after hospitalisation with AKI. Recruitment was well above the target, indicating that patients are willing to participate in studies monitoring kidney function after an episode of AKI. Learning from the workload of this study, we expect that one full time researcher would be required to recruit at the target rate of 5 patients per week, and complete the study data collection visits.

Retention was just below target, due to patient withdrawal and the competing risk of death. When determining the sample size for a longer term study on obesity associated outcomes after AKI it will be important to account for loss of follow up due to the competing risk of death, as 1 and 5 year mortality post AKI can be >40% and >60% respectively, in studies utilising healthcare data [30].

Timing of data collection and selection of study variables

The initial study visit 1 month post the peak creatinine was demonstrated not to be feasible, as only one quarter of patients were able to complete the baseline visit within this time frame. As in other prospective studies, performing the baseline study visit 3 months post the AKI episode is recommended [31, 32].

In this feasibility study, data were collected with a view to determining the optimal markers to select for measurement in larger prospective observational studies, and to formulate hypotheses for mechanisms of obesity associated outcomes after AKI. Waist circumference and conicity index correlated highly with BMI, however neither measure discriminated differently to BMI in associations with change in $eGFR_{creat}$, adipose tissue hormones, proteinuria or markers of

For peer review only - http://bmjopen.bmj.com/site/about/guidelines.xhtml

inflammation. Single measures of hormonal markers of adiposity post AKI, such as leptin and adiponectin, may not provide any additional mechanistic understanding over BMI alone. Together, these results indicate that BMI is an adequate classifier of obesity in this population. IL-6 and cystatin-C, as markers of inflammation and kidney damage respectively, are likely to provide insight into prediction of risk for CKD outcomes after AKI, as our feasibility data suggest that IL-6 is a more sensitive than hs-CRP.

For peer review only

Measurement of eGFR in obesity

The utility of prediction equations for eGFR in obesity remains unclear, particularly when body size or body composition is in flux [25, 33, 34]. Estimated $GFR_{creat+cysC}$ provided the closest estimate to measured GFR in obesity pre and post bariatric surgery [25], but has not been studied extensively post AKI. We chose to examine it as a potential measure of kidney function in this feasibility study. There appeared to be more undulation in eGFR with both equations in patients with obesity, regardless of underlying level of kidney function. This may be attributed to the known reduced accuracy of eGFR predictions equations with obesity [26], and highlights the importance of using multiple measures of eGFR over time when examining long term change in kidney function. Bland Altman plots did not indicate any difference in the bias between the 2 methods across categories of BMI but did suggest that at higher eGFR levels the estimating equation using cystatin generated lower results than the creatinine only equation. Additionally, there was no association between baseline BMI and cystatin C in this cohort, and together these may indicate that cystatin C was not influenced by obesity in this study. Further studies comparing different estimating equations to measured GFR in post AKI populations are required to determine which methods are valid.

For peer review only - http://bmjopen.bmj.com/site/about/guidelines.xhtml

At all time points post AKI, $eGFR_{creat}$ was higher, than $eGFR_{creat+cysC}$. Other studies have also found differences in creatinine and cystatin-C based equations in specific populations. In an elderly community population, smoking, age, BMI, inflammation (measured by C-reactive protein) and glucocorticoid were associated with higher $eGFR_{creat}$ and lower $eGFR_{cysC}$ [35]. The authors suggested that these differences could be due to a reduction in muscle mass and inflammation, respectively [35]. These non-GFR determinants of creatinine and cystatin-C are both also likely evident after AKI, and may explain the lower eGFRs observed in the current study with the inclusion of cystatin-C. Cystatin-C correlated with indices of adiposity, although in a previous study the difference between measured GFR and $eGFR_{cysC}$ did not correlate with body fat measured by DEXA in young males with normal kidney function [36]. In that study, $eGFR_{creat+cysC}$ performed best for both high lean mass and high fat mass groups, although the $eGFR_{creat}$ had greater accuracy for the normal BMI group. Together, these findings suggest that both body composition and inflammation are potential confounding factors for interpretation of estimates of kidney function post AKI, due to their non-GFR influences on creatinine and cystatin-C. Studies examining $eGFR_{creat+cysC}$ at 90 days post AKI as a risk factor for development or progression of CKD are warranted.

Development and progression of CKD

Almost one-third of patients showed development or progression of CKD 1 year post AKI. Horne and colleagues reported CKD development or progression in 24% of their prospective cohort at 3 years post AKI [8], whilst in those with complete recovery post AKI, development of stage 3 CKD occurred in 15% of patients after median follow up of 2.5 years [5]. 50% of the present cohort experienced stage 3 AKI, whilst most observational cohorts typically include 60-70% of patients with stage 1 AKI, [8, 37]. Almost half the cohort had existing CKD and more patients required renal replacement therapy during hospitalisation than in the other UK studies

For peer review only - http://bmjopen.bmj.com/site/about/guidelines.xhtml

[38], both of which are likely contributors to the high rate of CKD development and progression in the present study.

Raised IL-6 may be a risk factor associated with worsening of eGFR after AKI. One potential contributing factor could be a latent effect of obesity on inflammation, which may influence CKD development after AKI. Whether there is an interaction or "cross-talk" between obesity and markers of inflammation in CKD progression remains to be studied further in a suitably powered study with longer term follow up. Low BMI may also be associated with inflammation, so future studies should seek to determine if non-linear relationships are evident. This study was not designed to examine if the risk of CKD development or progression is higher with obesity, so the reported lack of difference between obese and non-obese groups could represent type II error. In addition to small sample size, we also recognise the limitation of short-term follow up of 1 year, and it is possible that risks and outcomes associated with obesity will be more pronounced over longer periods of time. In any fully powered study of CKD outcomes following AKI, other factors such as the severity and duration of AKI, previous or repeated AKI episodes, proteinuria, severity of the illness precipitating the AKI and subsequent interventions, frailty, and pre-existing CKD are all likely confounders to be considered when examining the risk of later CKD development or progression.

The tools available to identify AKI limit all studies on outcomes following AKI. Whilst the KDIGO definition of AKI refined previous criteria, it is still limited by changes in creatinine and urine output, both deemed late markers of kidney damage [28]. Furthermore, awareness of AKI risk may influence the frequency of measurement of serum creatinine, the use of an AKI identification algorithm which excludes urinary output identification criteria, and inclusion in studies such as this of only participants with established baseline kidney function, introduce bias in participant identification [30].

For peer review only - http://bmjopen.bmj.com/site/about/guidelines.xhtml

In conclusion, we have demonstrated that it is feasible to conduct a prospective study on the effect of obesity on the long term outcomes after AKI. Our results inform the design of future definitive studies, which should include measures of pre-AKI kidney function, long-term follow up after the acute illness and recovery phase, and careful consideration of the use of creatinine, cystatin-C, or measured GFR to assess kidney function.

a. Contributorship statement: HM, CM, and NS contributed to the conception and design of the study, HM, NS, GP, IM and SJ developed the methodology, HM, SJ, RB & KW collected and analysed the data, HM drafted the work and all authors revised it critically for important intellectual content. All authors approved of the final version submitted and agree to be accountable for all aspects of the work.

b. Competing interests: None of the authors have any competing interests to declare

c. Funding: This work was supported by a National Institute of Health Research (NIHR)/Higher Education England (HEE) Clinical Lectureship award (CAT CL-2014-05-005). This paper presents independent research funded by the National Institute for Health Research (NIHR) and Health Education England. The views expressed are those of the authors and not necessarily those of the NHS, the NIHR or the Department of Health.

d. data sharing statement:

The study protocol, informed consent form, clinical study report and individual participant data that underlie the results reported in this article, after deidentification (text, tables, figures and appendices) will be made available upon request, immediately after publication and ending 4 years after article publication. This information will be available to researchers who provide a methodologically sound proposal within the limits of the approval given by the original independent ethics review committee, or investigators whose proposed use of the data has been approved by another independent review committee ("learned intermediary") identified for this purpose, for

For peer review only - http://bmjopen.bmj.com/site/about/guidelines.xhtml

individual participant data meta-analysis. Proposals should be directed to helen.maclaughlin@nhs.net. To gain access, data requestors will need to sign a data access agreement. Proposals may be submitted up to 36 months following article publication.

Acknowledgements: We would like to thank Sarah Mackie, Rebecca Freeman, Richard Hull, Joble Joseph and Gurminder Khamba for their clinical expertise and assistance in identifying potential participants.

References

1. Mehta RL, Kellum JA, Shah SV *et al.* Acute Kidney Injury Network: report of an initiative to improve outcomes in acute kidney injury. Critical care 2007; 11: R31
2. Kidney Disease: Improving Global Outcomes (KDIGO) CKD Work Group. KDIGO 2012 Clinical practice guideline for the evaluation and management of chronic kidney disease. Kidney International Supplements 2013; 3: 1-150
3. NHS England. *Patient Safety Alert. Stage 3 Directive: Standardising the early indentification of Acute Kidney Injury*. Patient Safety Alert 2015 30 January 2015 [cited 2017 10 May]; Available from: https://www.england.nhs.uk/wp-content/uploads/2014/06/psa-aki-alg.pdf.
4. Selby NM, Crowley L, Fluck RJ *et al.* Use of electronic results reporting to diagnose and monitor AKI in hospitalized patients. Clin J Am Soc Nephrol 2012; 7: 533-540
5. Jones J, Holmen J, De Graauw J, Jovanovich A, Thornton S , Chonchol M. Association of complete recovery from acute kidney injury with incident CKD stage 3 and all-cause mortality. Am J Kidney Dis 2012; 60: 402-408
6. Schmitt R, Coca S, Kanbay M, Tinetti ME, Cantley LG , Parikh CR. Recovery of kidney function after acute kidney injury in the elderly: a systematic review and meta-analysis. Am J Kidney Dis 2008; 52: 262-271
7. Venkatachalam MA, Griffin KA, Lan R, Geng H, Saikumar P , Bidani AK. Acute kidney injury: a springboard for progression in chronic kidney disease. Am J Physiol Renal Physiol 2010; 298: F1078-1094
8. Horne KL, Packington R, Monaghan J, Reilly T , Selby NM. Three-year outcomes after acute kidney injury: results of a prospective parallel group cohort study. BMJ Open 2017; 7: e015316
9. Coca SG, Singanamala S , Parikh CR. Chronic kidney disease after acute kidney injury: a systematic review and meta-analysis. Kidney Int 2012; 81: 442-448
10. Sawhney S, Marks A, Fluck N, Levin A, Prescott G , Black C. Intermediate and Long-term Outcomes of Survivors of Acute Kidney Injury Episodes: A Large Population-Based Cohort Study. Am J Kidney Dis 2016
11. Sawhney S, Mitchell M, Marks A, Fluck N , Black C. Long-term prognosis after acute kidney injury (AKI): what is the role of baseline kidney function and recovery? A systematic review. BMJ Open 2015; 5: e006497
12. Fox CS, Larson MG, Leip EP, Culleton B, Wilson PW , Levy D. Predictors of new-onset kidney disease in a community-based population. Journal of the American Medical Association 2004; 291: 844-850
13. Munkhaugen J, Lydersen S, Wideroe TE , Hallan S. Prehypertension, obesity, and risk of kidney disease: 20-year follow-up of the HUNT I study in Norway. American Journal of Kidney Diseases 2009; 54: 638-646
14. Hsu C, McCulloch CE, Iribarren C, Darbinian J , Go ASW. Body Mass Index and Risk for End-Stage Renal Disease. Ann Intern Med 2006; 144: 21-28

For peer review only - http://bmjopen.bmj.com/site/about/guidelines.xhtml

15. Gelber RP, Kurth T, Kausz AT *et al.* Association Between Body Mass Index and CKD in Apparently Healthy Men. American Journal of Kidney Diseases 2005; 46: 871-880
16. Hallan S, de Mutsert R, Carlsen S, Dekker FW, Aasarød K , Holmen J. Obesity, Smoking, and Physical Inactivity as Risk Factors for CKD: Are Men More Vulnerable? American Journal of Kidney Diseases 2006; 47: 396-405
17. Druml W, Metnitz B, Schaden E, Bauer P , Metnitz PG. Impact of body mass on incidence and prognosis of acute kidney injury requiring renal replacement therapy. Intensive Care Med 2010; 36: 1221-1228
18. Soto GJ, Frank AJ, Christiani DC , Gong MN. Body mass index and acute kidney injury in the acute respiratory distress syndrome. Crit Care Med 2012; 40: 2601-2608
19. Shashaty MG, Meyer NJ, Localio AR *et al.* African American race, obesity, and blood product transfusion are risk factors for acute kidney injury in critically ill trauma patients. J Crit Care 2012; 27: 496-504
20. Shen WW, Chen HM, Chen H, Xu F, Li LS , Liu ZH. Obesity-Related Glomerulopathy: Body Mass Index and Proteinuria. Clinical Journal of the American Society of Nephrology 2010; 5: 1401-1409
21. Levey AS, Bosch JP, Lewis JB, Greene T, Rogers N , Roth D. A more accurate method to estimate glomerular filtration rate from serum creatinine: a new prediction equation. Modification of Diet in Renal Disease Study Group. Ann Intern Med 1999; 130: 461-470
22. Delanaye P, Radermecker RP, Rorive M, Depas G , Krzesinski JM. Indexing glomerular filtration rate for body surface area in obese patients is misleading: concept and example. Nephrology Dialysis Transplantation 2005; 20: 2024-2028
23. Levey AS, Stevens LA, Schmid CH *et al.* A new equation to estimate glomerular filtration rate. Ann Intern Med 2009; 150: 604-612
24. Inker LA, Schmid CH, Tighiouart H *et al.* Estimating glomerular filtration rate from serum creatinine and cystatin C. N Engl J Med 2012; 367: 20-29
25. Friedman AN, Moe S, Fadel WF *et al.* Predicting the Glomerular Filtration Rate in Bariatric Surgery Patients. Am J Nephrol 2013; 39: 8-15
26. Lemoine S, Guebre-Egziabher F, Sens F *et al.* Accuracy of GFR Estimation in Obese Patients. Clin J Am Soc Nephrol 2014; 9: 720-727
27. Reeves GK, Balkwill A, Cairns BJ, Green J , Beral V. Hospital admissions in relation to body mass index in UK women: a prospective cohort study. BMC Med 2014; 12: 45
28. Kellum JA, Lameire N, Aspelin P *et al.* Kidney disease: Improving global outcomes (KDIGO) acute kidney injury work group. KDIGO clinical practice guideline for acute kidney injury. Kidney International Supplements 2012; 2: 1-138
29. World Health Organisation. *BMI classification.* 2006 [cited 2010 11 June]; Available from: http://apps.who.int/bmi/index.jsp?introPage=intro_3.html.
30. Sawhney S, Fluck N, Fraser SD *et al.* KDIGO-based acute kidney injury criteria operate differently in hospitals and the community-findings from a large population cohort. Nephrol Dial Transplant 2016; 31: 922-929
31. Go AS, Parikh CR, Ikizler TA *et al.* The assessment, serial evaluation, and subsequent sequelae of acute kidney injury (ASSESS-AKI) study: design and methods. BMC nephrology 2010; 11: 22
32. Horne KL, Shardlow A, Taal MW , Selby NM. Long Term Outcomes after Acute Kidney Injury: Lessons from the ARID Study. Nephron 2015; 131: 102-106
33. Lieske JC, Collazo-Clavell ML, Sarr MG, Rule AD, Bergstralh EJ , Kumar R. Gastric bypass surgery and measured and estimated GFR in women. Am J Kidney Dis 2014; 64: 663-665
34. Chang AR, Zafar W , Grams ME. Kidney Function in Obesity-Challenges in Indexing and Estimation. Adv Chronic Kidney Dis 2018; 25: 31-40

For peer review only - http://bmjopen.bmj.com/site/about/guidelines.xhtml

35. Legrand H, Werner K, Christensson A, Pihlsgard M , Elmstahl S. Prevalence and determinants of differences in cystatin C and creatinine-based estimated glomerular filtration rate in community-dwelling older adults: a cross-sectional study. BMC Nephrol 2017; 18: 350
36. Chew-Harris JS, Florkowski CM, George PM, Elmslie JL , Endre ZH. The relative effects of fat versus muscle mass on cystatin C and estimates of renal function in healthy young men. Annals of clinical biochemistry 2013; 50: 39-46
37. Selby NM, Kolhe NV, McIntyre CW *et al.* Defining the cause of death in hospitalised patients with acute kidney injury. PLoS One 2012; 7: e48580
38. Horne KL, Packington R, Monaghan J, Reilly T, McIntyre CW , Selby NM. The effects of acute kidney injury on long-term renal function and proteinuria in a general hospitalised population. Nephron Clin Pract 2014; 128: 192-200

Figure Legends

Figure 1: Recruitment Flow Diagram

Figure 2: estimated glomerular filtration rate ($eGFR_{creat}$) from pre AKI to 12 months post AKI by BMI category with and without pre-existing CKD

Figure 3: estimated glomerular filtration rate ($eGFR_{creat+cysC}$) from baseline to 12 months post AKI by BMI category with and without pre-existing CKD

Figure 4: Bland Altman plot of mean of individual $eGFR_{creat}$ and $eGFR_{creat+cysC}$ versus difference for the normal weight group (body mass index (BMI) <25 kg/m^2); horizontal lines represent mean difference and 95% limits of agreement

Figure 5: Bland Altman plot of mean of individual $eGFR_{creat}$ and $eGFR_{creat+cysC}$ versus difference for the overweight weight group (body mass index (BMI) 25-29.9 kg/m^2); horizontal lines represent mean difference and 95% limits of agreement

Figure 6: Bland Altman plot of mean of individual $eGFR_{creat}$ and $eGFR_{creat+cysC}$ versus difference for the obese group (body mass index (BMI) 30+ kg/m^2); horizontal lines represent mean difference and 95% limits of agreement

For peer review only

For peer review only - http://bmjopen.bmj.com/site/about/guidelines.xhtml

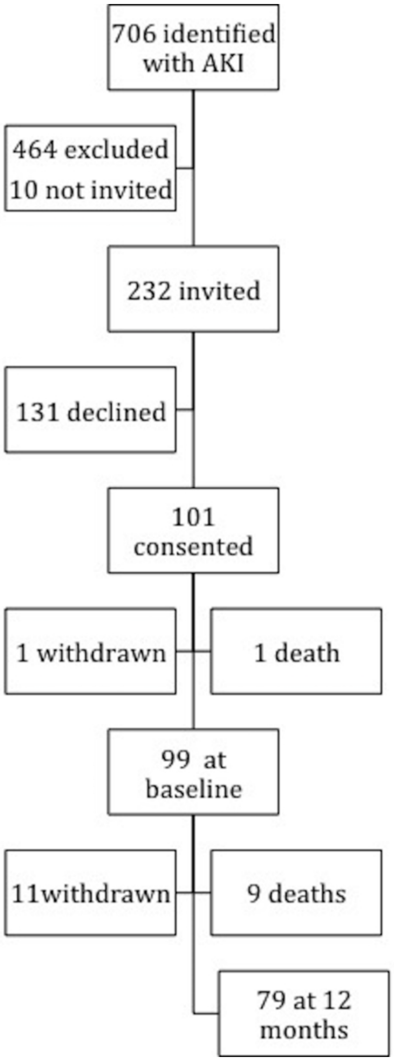

Figure 1: Recruitment Flow Diagram

192x202mm (300 x 300 DPI)

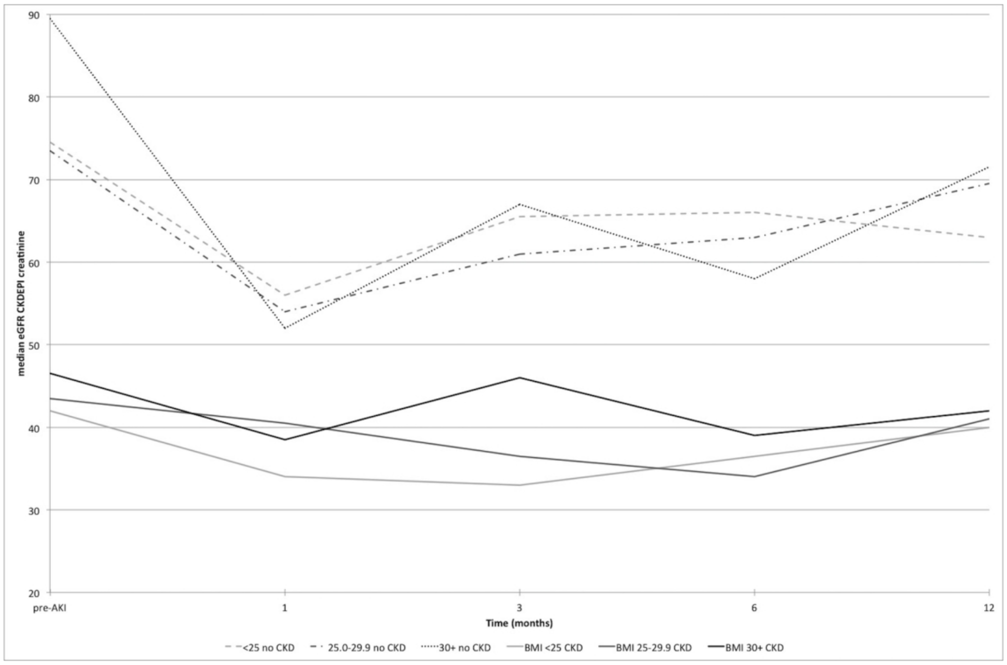

Figure 2: estimated glomerular filtration rate ($eGFR_{creat}$) from pre AKI to 12 months post AKI by BMI category with and without pre-existing CKD

150x98mm (300 x 300 DPI)

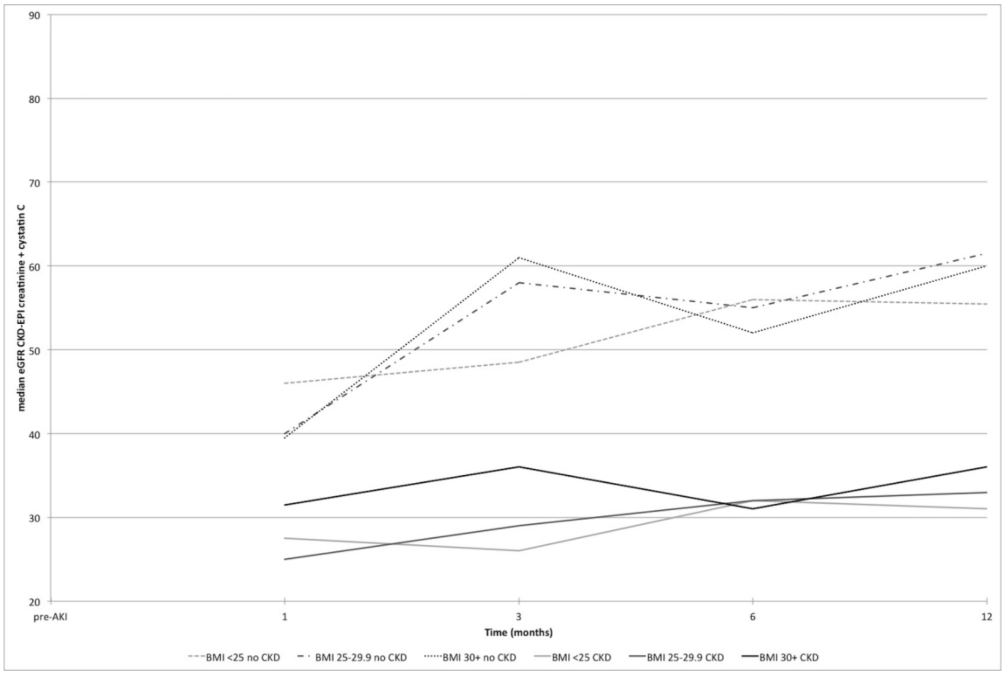

Figure 3: estimated glomerular filtration rate ($eGFR_{creat+cysC}$) from baseline to 12 months post AKI by BMI category with and without pre-existing CKD

150x100mm (300 x 300 DPI)

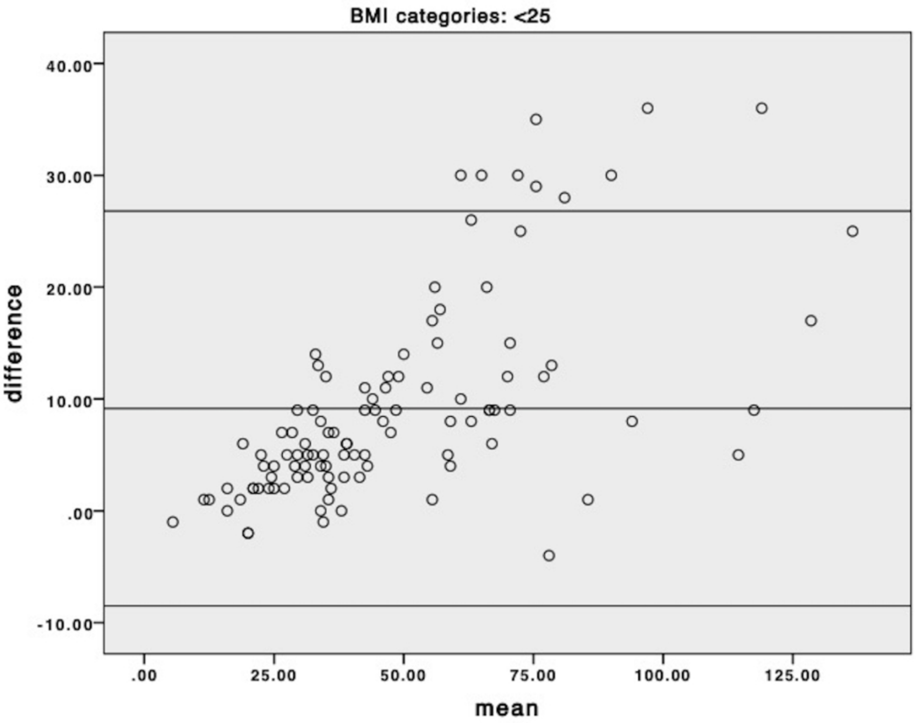

Figure 4: Bland Altman plot of mean of individual $eGFR_{creat}$ and $eGFR_{creat+cysC}$ versus difference for the normal weight group (body mass index (BMI) <25 kg/m2); horizontal lines represent mean difference and 95% limits of agreement

112x90mm (300 x 300 DPI)

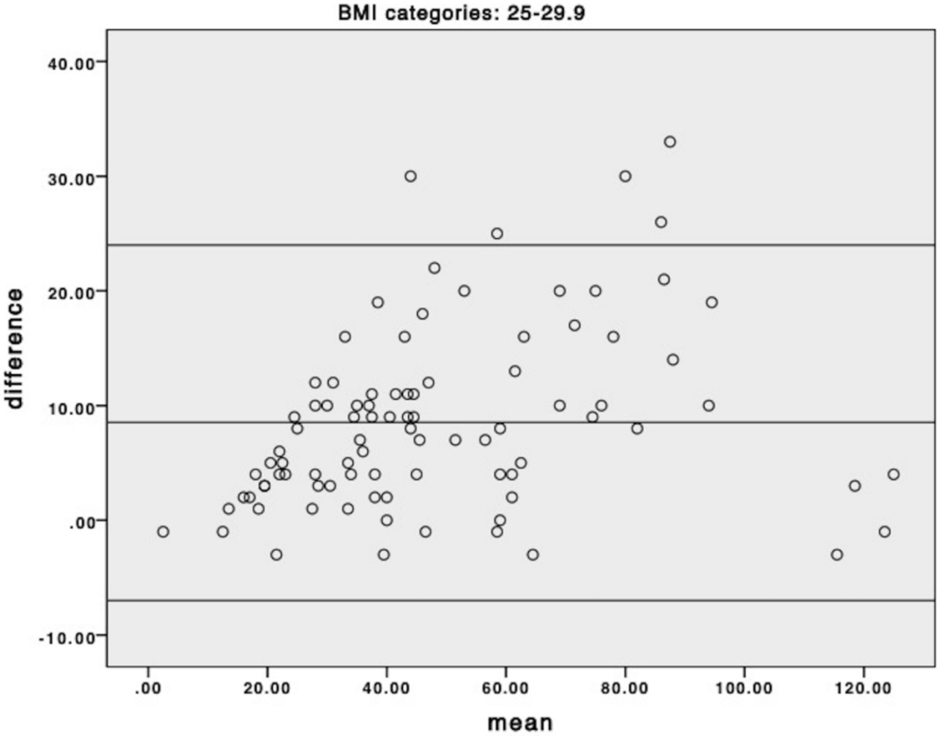

Figure 5: Bland Altman plot of mean of individual $eGFR_{creat}$ and $eGFR_{creat+cysC}$ versus difference for the overweight weight group (body mass index (BMI) 25-29.9 kg/m2); horizontal lines represent mean difference and 95% limits of agreement

113x90mm (300 x 300 DPI)

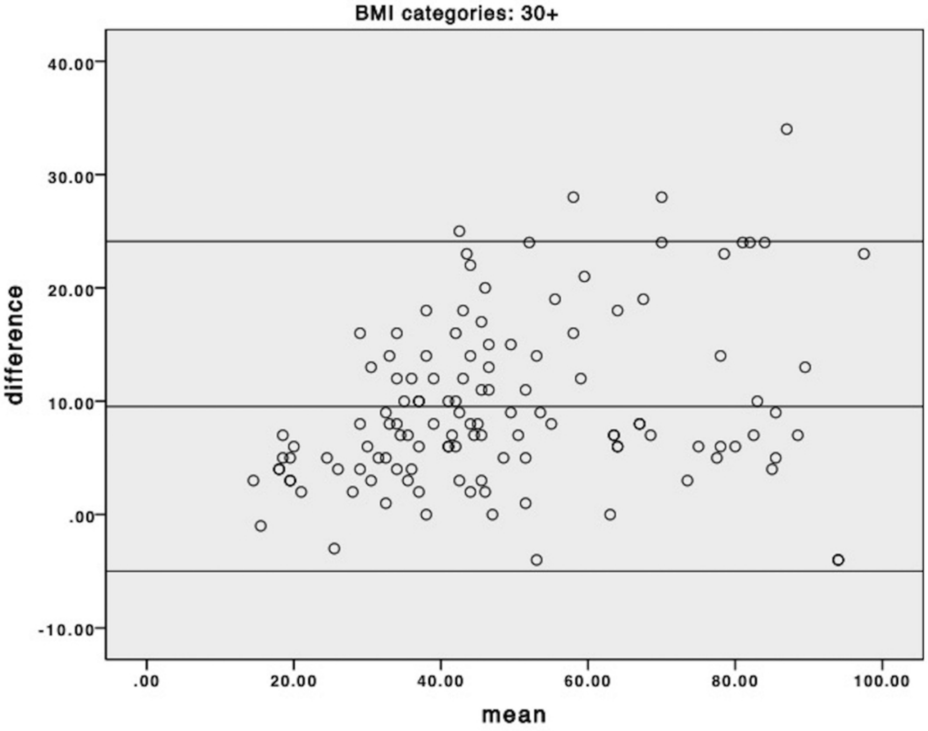

Figure 6: Bland Altman plot of mean of individual $eGFR_{creat}$ and $eGFR_{creat+cysC}$ versus difference for the obese group (body mass index (BMI) 30+ kg/m2); horizontal lines represent mean difference and 95% limits of agreement

115x90mm (300 x 300 DPI)

STROBE 2007 (v4) Statement—Checklist of items that should be included in reports of *cohort studies*

Section/Topic	Item #	Recommendation	Reported on page #
**Title and abstract**	1	(*a*) Indicate the study's design with a commonly used term in the title or the abstract	1
		(*b*) Provide in the abstract an informative and balanced summary of what was done and what was found	2
**Introduction**			
Background/rationale	2	Explain the scientific background and rationale for the investigation being reported	3-4
Objectives	3	State specific objectives, including any prespecified hypotheses	4
**Methods**			
Study design	4	Present key elements of study design early in the paper	5
Setting	5	Describe the setting, locations, and relevant dates, including periods of recruitment, exposure, follow-up, and data collection	5-6
Participants	6	(*a*) Give the eligibility criteria, and the sources and methods of selection of participants. Describe methods of follow-up	5-6
		(*b*) For matched studies, give matching criteria and number of exposed and unexposed	n/a
Variables	7	Clearly define all outcomes, exposures, predictors, potential confounders, and effect modifiers. Give diagnostic criteria, if applicable	7
Data sources/ measurement	8*	For each variable of interest, give sources of data and details of methods of assessment (measurement). Describe comparability of assessment methods if there is more than one group	6-7
Bias	9	Describe any efforts to address potential sources of bias	n/a
Study size	10	Explain how the study size was arrived at	5
Quantitative variables	11	Explain how quantitative variables were handled in the analyses. If applicable, describe which groupings were chosen and why	6
Statistical methods	12	(*a*) Describe all statistical methods, including those used to control for confounding	7
		(*b*) Describe any methods used to examine subgroups and interactions	7
		(*c*) Explain how missing data were addressed	7
		(*d*) If applicable, explain how loss to follow-up was addressed	
		(*e*) Describe any sensitivity analyses	n/a
**Results**			

For peer review only

Participants	13*	(a) Report numbers of individuals at each stage of study—eg numbers potentially eligible, examined for eligibility, confirmed eligible, included in the study, completing follow-up, and analysed	8
		(b) Give reasons for non-participation at each stage	Fig 1 and 9
		(c) Consider use of a flow diagram	Fig 1
Descriptive data	14*	(a) Give characteristics of study participants (eg demographic, clinical, social) and information on exposures and potential confounders	8-9
		(b) Indicate number of participants with missing data for each variable of interest	10
		(c) Summarise follow-up time (eg, average and total amount)	10
Outcome data	15*	Report numbers of outcome events or summary measures over time	Table 3
Main results	16	(*a*) Give unadjusted estimates and, if applicable, confounder-adjusted estimates and their precision (eg, 95% confidence interval). Make clear which confounders were adjusted for and why they were included	11
		(*b*) Report category boundaries when continuous variables were categorized	11
		(*c*) If relevant, consider translating estimates of relative risk into absolute risk for a meaningful time period	n/a
Other analyses	17	Report other analyses done—eg analyses of subgroups and interactions, and sensitivity analyses	11-12
**Discussion**			
Key results	18	Summarise key results with reference to study objectives	13, 16
**Limitations**			
Interpretation	20	Give a cautious overall interpretation of results considering objectives, limitations, multiplicity of analyses, results from similar studies, and other relevant evidence	14-16
Generalisability	21	Discuss the generalisability (external validity) of the study results	
**Other information**			
Funding	22	Give the source of funding and the role of the funders for the present study and, if applicable, for the original study on which the present article is based	17

*Give information separately for cases and controls in case-control studies and, if applicable, for exposed and unexposed groups in cohort and cross-sectional studies.

Note: An Explanation and Elaboration article discusses each checklist item and gives methodological background and published examples of transparent reporting. The STROBE checklist is best used in conjunction with this article (freely available on the Web sites of PLoS Medicine at http://www.plosmedicine.org/, Annals of Internal Medicine at http://www.annals.org/, and Epidemiology at http://www.epidem.com/). Information on the STROBE Initiative is available at www.strobe-statement.org.

For peer review only
